# Disordered protein-graphene oxide co-assembly and supramolecular biofabrication of functional fluidic devices

**DOI:** 10.1038/s41467-020-14716-z

**Published:** 2020-03-04

**Authors:** Yuanhao Wu, Babatunde O. Okesola, Jing Xu, Ivan Korotkin, Alice Berardo, Ilaria Corridori, Francesco Luigi Pellerej di Brocchetti, Janos Kanczler, Jingyu Feng, Weiqi Li, Yejiao Shi, Vladimir Farafonov, Yiqiang Wang, Rebecca F. Thompson, Maria-Magdalena Titirici, Dmitry Nerukh, Sergey Karabasov, Richard O. C. Oreffo, Jose Carlos Rodriguez-Cabello, Giovanni Vozzi, Helena S. Azevedo, Nicola M. Pugno, Wen Wang, Alvaro Mata

**Affiliations:** 1 Institute of Bioengineering, Queen Mary University of London, London, E1 4NS UK; 2 School of Engineering and Materials Science, Queen Mary University of London, London, E1 4NS UK; 3 School of Pharmacy, University of Nottingham, NG7 2RD Nottingham, UK; 4 Department of Chemical and Environmental Engineering, University of Nottingham, NG7 2RD Nottingham, UK; 5 Biodiscovery Institute, University of Nottingham, NG7 2RD Nottingham, UK; 6 Laboratory of Bio-inspired, Bionic, Nano, Meta Materials & Mechanics, Università di Trento, via Mesiano, 77, I-38123 Trento, Italy; 7 Research Center‘E. Piaggio’ & Dipartimento di Ingegneria dell’Informazione, University of Pisa, Largo Lucio Lazzarino, 256126 Pisa, Italy; 8 Bone and Joint Research Group, Centre for Human Development, Stem Cells and Regeneration, Institute of Developmental Sciences, University of Southampton, Southampton, SO16 6YD UK; 9 Department of Physical Chemistry, V. N. Karazin Kharkiv National University, Svobody Sq. 4, Kharkiv, 61022 Ukraine; 10 United Kingdom Atomic Energy Authority, Culham Science Centre, Abingdon, OX14 3DB UK; 11 The Astbury Biostructure Laboratory, Astbury Centre for Structural Molecular Biology, Faculty of Biological Sciences, University of Leeds, Leeds, UK; 12 Systems Analytics Research Institute, Department of Mathematics, Aston University, Birmingham, B4 7ET UK; 13 BIOFORGE Group, University of Valladolid, CIBER-BBN, 47011 Valladolid, Spain; 14 KET Labs, Edoardo Amaldi Foundation, Via del Politecnico snc, 00133 Rome, Italy; 15 Present Address: Mathematical Sciences, University of Southampton, Southampton SO17 1BJ, UK; 16 Present Address: C3A - Center Agriculture Food Environment, University of Trento/Fondazione Edmund Mach, Via Edmund Mach, 1 - 38010, San Michele allʼAdige (TN), Italy

**Keywords:** Biomaterials - proteins, Graphene, Mechanical and structural properties and devices

## Abstract

Supramolecular chemistry offers an exciting opportunity to assemble materials with molecular precision. However, there remains an unmet need to turn molecular self-assembly into functional materials and devices. Harnessing the inherent properties of both disordered proteins and graphene oxide (GO), we report a disordered protein-GO co-assembling system that through a diffusion-reaction process and disorder-to-order transitions generates hierarchically organized materials that exhibit high stability and access to non-equilibrium on demand. We use experimental approaches and molecular dynamics simulations to describe the underlying molecular mechanism of formation and establish key rules for its design and regulation. Through rapid prototyping techniques, we demonstrate the system’s capacity to be controlled with spatio-temporal precision into well-defined capillary-like fluidic microstructures with a high level of biocompatibility and, importantly, the capacity to withstand flow. Our study presents an innovative approach to transform rational supramolecular design into functional engineering with potential widespread use in microfluidic systems and organ-on-a-chip platforms.

## Abstract (web summary)

Self-organising systems have huge potential in device design and fabrication; however, demonstrations of this are limited. Here, the authors report on a combination of disordered proteins and graphene oxide which allows spatio-temporal patterning and demonstrate the fabrication of microfluidic devices.

## Introduction

There is an increasing interest to generate materials with bioinspired functions^1^, such as the capacity to grow^2^, self-replicate^3^, or controllably respond to specific stimuli^4^. Biological materials acquire most of these functionalities as a consequence of their ability to self-assemble various types of building blocks at multiple length scales. Engineering materials in this manner provides an opportunity to take advantage of the individual building blocks while enabling emergent properties as a result of their interactions^5,6^. Consequently, multicomponent self-assembly represents an attractive route to develop more complex materials^7^ with enhanced modularity and tuneability of properties^8,9^, such as structural hierarchy^10^, adhesion^11^, electrical conductivity^12^, or the capacity to grow^13^.

Proteins are the most functional building blocks of organisms^14^ and, as such, have been thoroughly explored to engineer intelligent materials^15–17^. However, new discoveries are shaping our understanding of how proteins function and providing new insights for their utilization. For example, there is increasing evidence that both ordered (i.e., β-sheet and α-helix) and disordered (i.e., random coil) regions of proteins play a role in their functionality^18^ and growing acceptance that this functionality is regulated by their interaction with other molecules^19^. Based on these principles, proteins are emerging as dynamic building blocks of multicomponent systems to engineer intelligent materials. We have recently reported on the possibility to exploit the disordered nature of elastin-like recombinamers (ELRs) to modulate their conformation and generate dynamic^13^ or hierarchically mineralizing^20^ materials. ELRs, also known as the recombinantly produced elastin-like polypeptides, are based on the natural elastin motif Val-Pro-Gly-X-Gly (VPGXG), where X could be any amino acid apart from proline^21^. These molecules exhibit a reversible-phase transition with a change in temperature and have been used to create biocompatible materials^22^.

Multicomponent self-assembly also offers a unique opportunity to engineer complex hybrid systems. In particular, the controlled incorporation of graphene as a building-block could lead to the design of new biomaterials that benefit from its distinctive two-dimensional (2D) structure and outstanding electronic, thermal, and mechanical properties^23–25^. Toward this goal, graphene and its derivatives have been modified with biomacromolecules^26^, such as DNA^27^, proteins^28^, and biopolymers^29^ and used in, for example, implants and scaffolds for cell culture and regenerative medicine^30,31^. Furthermore, graphene oxide (GO) is gaining significant interest and being used instead of graphene given its rich oxygen-containing functional groups (hydroxyl, epoxy, carbonyl, and carboxyl), which facilitate designed interactions with different molecules. However, both graphene and GO exhibit key limitations such as dose-dependent toxicity and issues associated with hierarchical organization and the ability to generate uniform and stable structures^24–26^.

Material platforms that exploit the functionalities of both proteins and GO and enable their multiscale organization offer exciting possibilities for the engineering of advanced materials. We report a hierarchical self-assembling system that takes advantage of protein disorder-to-order transitions and supramolecular protein–GO interactions to enable both stable structures and access to non-equilibrium with spatial control to design functional materials and devices. Experimental approaches and molecular dynamics (MD) simulations were used to elucidate the underlying molecular mechanism and develop rules for its use. We show that the material can be combined with rapid-prototyping techniques to assemble well-defined tubular microstructures embedded with cells and into fluidic devices. The study introduces an innovative way to biofabricate by self-assembly complex and functional devices such as microfluidic systems or organ-on-a-chip devices.

## Results

### System rationale

Previous studies have demonstrated the possibility to co-assemble peptides with large macromolecules to generate hierarchical membranes at a liquid–liquid interface^10,13^. These systems rely on both molecular interactions, such as electrostatic and hydrophobic forces between the two components as well as their respective individual properties, such as molecular weight, charge, and 3D conformation. In particular, by co-assembling ELRs and peptide amphiphiles (PAs), we have previously reported on a diffusion–reaction mechanism that relies on PA diffusion to give rise to a multilayer membrane that can access non-equilibrium^13^. However, this material is fragile and can only be assembled in and is stable under a narrow window of environmental conditions (pH, temperature, and salt concentration), which limits its functionality and widespread use. Giving the need for multicomponent approaches that can turn molecular design into functional systems^1^, we envision the possibility to exploit the inherent properties of GO to work synergistically with disordered proteins to create materials with both emergent properties and functionality. Specifically, we reasoned that, unlike the PAs, the GO lamella conformation in aqueous environments^32^ would provide a supramolecular framework with high surface area for ELR interaction, reaching a level of integration far beyond that of the ELR-PA system. Furthermore, the GO’s flat-sheet organization at air–liquid interfaces^33^ would facilitate the generation of a diffusion–reaction process that takes advantage of the disordered nature of ELRs to diffuse, conform, and integrate with GO, generating a hierarchical process of assembly that can be manipulated on demand. In this way, we hypothesized that the co-assembly of GO and ELRs would lead to a robust supramolecular system where both strong molecular interactions and controllable access to non-equilibrium would lead to new materials with enhanced complexity and functionalities.

### Materials rationale

**Fig. 1:**
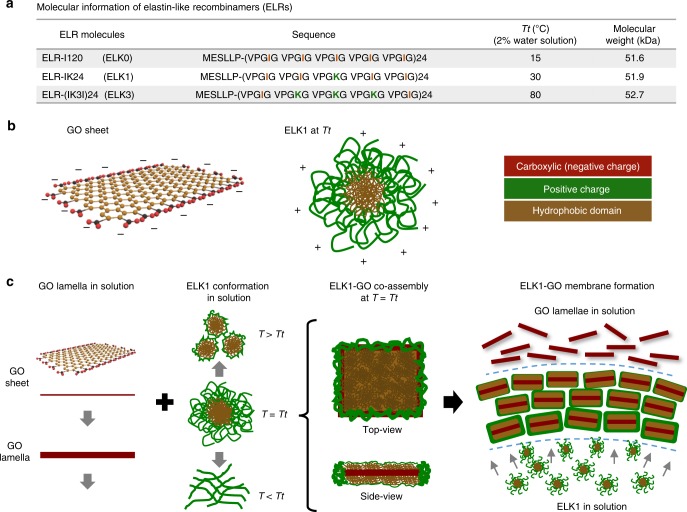
Molecular building blocks and rationale for co-assembly. **a** Table summarizes the key information of the three elastin-like recombinamers (ELRs) used in the study comprising similar molecular weight but different levels of hydrophobicity (VPGIG) and positive charge (VPGKG). **b** Illustrations of the molecular structure of a GO sheet and the supramolecular organization of ELK1 at its transition temperature (*Tt*) (30 °C) indicating both the charged (red and green) and hydrophobic (brown) segments. **c** Schematic of the proposed mechanism of formation illustrating the molecular and supramolecular conformation of the GO and ELK1 before and after co-assembly at the ELK1’s *Tt* as well as their interaction for membrane formation.

We used GO sheets of two different average lateral sizes, including larger GO (GO-L) measuring 10.5 ± 4.5 µm and smaller GO (GO-S) of 2.3 ± 0.9 µm, both exhibiting a typical hydrophobic surface and negatively charged carboxylic groups on their periphery (Fig. 1b and Supplementary Fig. 1). We chose ELRs as the protein component because of their modular and disordered nature^34^ and the possibility to exhibit different molecular conformations at different temperatures^35^. The ELK1 sequence (Fig. 1a) is a 51.9 kDa molecule consisting of 24 repeats of a single block made of four hydrophobic pentapeptides (VPGIG) and a positively charged (VPGKG) one. This relatively simple molecular design offers an accessible transition temperature (*Tt*) of 30 °C (at 2% ELK1 in MilliQ water) with clearly different ELR conformations above or below it, as well as medium molecular weight to enable both cooperative interactions between its charged and hydrophobic segments as well as with the anionic edge and hydrophobic surface of the GO (Fig. 1c). ELRs with similar molecular weight but different levels of charge and hydrophobicity (Fig. 1a), as well as a single repeat of an individual block of each of these three ELRs, were used as controls (Supplementary Figs. 2–4).

### Co-assembly

**Fig. 2:**
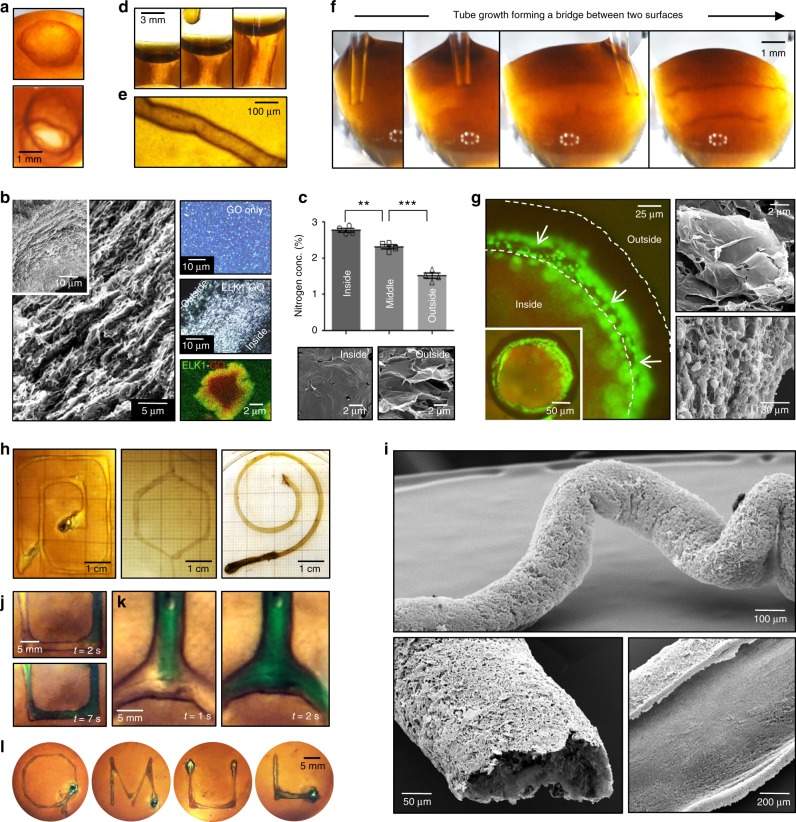
Co-assembly, structure, properties, and biofabrication of the ELK1–GO system. **a** Time-lapse images illustrate the dynamic properties of the ELK1–GO membrane first (**a**-Top forming a closed sac when a drop of ELK1 solution is immersed in a larger GO solution and second (**a**-Bottom) opening upon touching an interface within the first seconds of formation. **b** The membrane exhibits a multi-layered architecture of about 50 μm thick comprising aligned GO sheets throughout (birefringence inset) interacting with ELK1 molecules (fluorescence image, green: ELK1, red: GO), **c** which are observed to decrease in concentration from the inside to the outside as evidenced by wavelength-dispersive spectroscopy (WDS). Only ELK1 comprises nitrogen in its molecular structure. ±s.d. for *n* = 3. *p < 0.05. *t* test. **d** The system enables growing the membranes into longer tubes on demand by displacing an interface. **e** The robustness of the system enables formation of capillaries down to about 50 μm in internal diameter with 10 μm thick walls, **f** bridging of surfaces simply by touching two interfaces while injecting one solution into the other, and **g** co-assembling in salt solutions, opening the possibility to embed cells (green identified by white arrows) within the membrane (outlined by dashed lines) as the tubes are formed. The images are taken after 24 h of culture and correspond to a live (green)/dead (red) assay. Scanning electron micrographs of cells embedded within layers of GO (top) and a cross-section of the ELK1–GO membrane comprising cells within different layers (bottom). **h**–**l** Images demonstrate the versatility of the co-assembly system by incorporating it with 3D printing to fabricate well-defined fluidic devices consisting of high-aspect ratio tubular structures (**h**) of different internal diameters and comprising curves, angles of different sizes, and bifurcations (**h**, **i**, **l**) capable of withstanding flow within a few minutes of formation (**j**, **k**).

When an ELK1 solution at its *Tt* (30 °C) is immersed in a larger volume of a GO solution, a multilayered membrane of up to 50 µm in thickness develops at the interface around the immersed drop maintaining both solutions separated (Fig. 2a and Supplementary Movie 1). This membrane consists of layers made from both GO sheets and ELK1 (Fig. 2b, confocal), with GO sheets being present throughout the cross-section of the membrane (Fig. 2b, SEM) and ELK1 gradually decreasing in concentration from the inside (ELK1 side) to the outside (GO side) of the membrane (Fig. 2c and Supplementary Fig. 5). Multilayered structures are known to emerge from diffusion–reaction mechanisms^36^. We have previously demonstrated that co-assembling PAs with ELRs, it is possible to trigger a diffusion–reaction mechanism, which generates multilayered membranes capable of exhibiting dynamic properties^13^. Similarly, by touching any surface within the first few seconds of formation, the ELK1–GO membrane adheres, spontaneously and reproducibly opens, and can be manipulated to grow into tubular structures with spatiotemporal control (Fig. 2a, d, and Supplementary Movie 2). However, in this case, the underlying ELR-GO mechanism of interaction and supramolecular assembly lead to the growth of a material with remarkably enhanced properties.

### Material structure, properties, and biofabrication of devices

First, the ELK1–GO membrane is both dynamic and highly stable, permitting controlled anisotropic growth of tubular geometries that exhibit no apparent effects on their multilayered structure when the temperature drops below (down to 4 °C) or raises above (up to 70 °C) the *Tt* of ELK1. This enhanced stability is also evidenced by the capability to co-assemble capillary-like structures down to ~50 µm in internal diameter, defined by the size of the injecting tip. Moreover, the system also enables the capacity to decrease the thickness of the wall down to ~10 µm, achieved by removing the GO solution in order to stop the assembly after ~2 min (Fig. 2e). While smaller diameters may be possible by using smaller tips, wall-thicknesses below ~10 µm were found to be too fragile to be manipulated. In addition, these structures can be grown as tubular bridges between gaps by simply touching, adhering, opening, and sealing to a surface soon after co-assembly and continuing injecting ELK1 solution into the GO solution until the next surface is touched (Fig. 2f and Supplementary Movie 3). Moreover, the system works in salt-containing solutions such as cell culture media, which enables co-assembly and growth of capillary-like structures in the presence of cells, resulting in structures comprising cells embedded within and on the wall of the tube (Fig. 2g). Furthermore, given this versatility and robustness, we demonstrated the possibility to use rapid-prototyping techniques to guide the co-assembly process using an extrusion-based 3-D printer to print the ELK1 solution within a GO solution (Supplementary Movie 4), generating fluidic devices containing high-aspect ratio tubular structures of different internal diameters and comprising curves (Fig. 2h, i and Supplementary Movie 5), angles of different sizes (Fig. 2h, i), and bifurcations (Fig. 2h, k and Supplementary Movie 6). The fluidic devices were able to withstand aqueous flows of up to 12.5 mL/min for at least 24 h and within 60 min of formation (Supplementary Movies 7 and 8). The highest flows would generate 0.26 N/m^2^ shear stress, which is within the range of mean shear stress values observed in common carotid arteries (0.7 N/m^2^)^37^. Altogether, these capabilities suggest that the mechanism of formation exhibits both strong ELK1–GO interactions at the molecular scale and integrated organization at higher size scales (Supplementary Fig. 6 (ELK1)).

### Underlying molecular mechanism of assembly: ELK1–GO molecular interactions

**Fig. 3:**
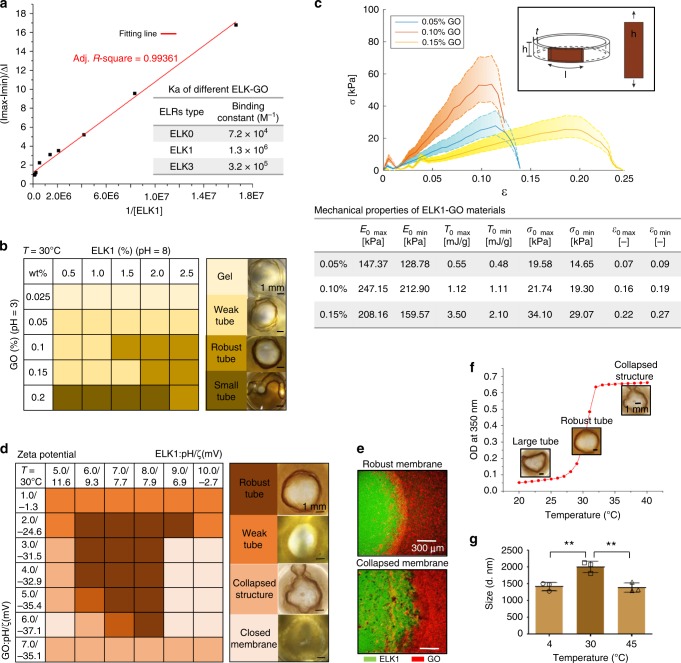
Molecular interaction, composition, and mechanical properties of ELR–GO. **a** Binding constants (Ka) for the different ELR-GO combinations calculated by a Benesi–Hildebrand equation based on fluorescence emission titration of a mixture of GO (2.5 × 10^−3^ wt%) in MilliQ water solution and increasing concentrations of ELRs revealing higher Ka for ELK1–GO compared to ELK0–GO and ELK3–GO. **b** Table illustrating the role of building-block concentration ratio on the formation of ELK1–GO tubes. **c** Nanotensile test results described with the Weibull distribution and reported in the table reveal that the strength, the strain at break, and the toughness modulus increased on tubes formed with increasing concentrations of GO but the elastic modulus was highest on tubes made with medium level (0.10%) GO compared to lower (0.05%) and higher (0.15%) amounts. **d** Table illustrating the role of pH and ζ on the formation of the ELK1–GO tubes and their respective geometry. **e** Representative confocal microscopy qualitatively depicting the interface between ELK1 (green) and GO (red) during tube formation with different levels of definition as characterized in **d**. **f** Red line of the graph shows the turbidity changes of an ELK1 (2 wt%) solution in MilliQ water while inserted images depict the definition of tubes formed at specific temperatures. **g** Dynamic light scattering (DLS) revealing the presence of larger ELK1–GO aggregates at 30 °C compared to 4 °C and 45 °C. Error bars represent ±s.d. for *n* = 3. *p < 0.05. One-way ANOVA.

We first tested the presence of both electrostatic and hydrophobic forces by quantifying ELK–GO-binding constants using ELRs with varying levels of charge and hydrophobicity. Tubes formed on application of ELK1 and ELK3 but not ELK0, confirming the need for electrostatic forces for its assembly. Interestingly, the highest binding constant (Ka), calculated by fluorescence emission titration, was obtained with ELK1 (1.3 × 10^6^) compared to ELK0 (7.2 × 10^4^) and ELK3 (3.2 × 10^5^) (Fig. 3a, Supplementary Fig. 6b, c). Using this method, we also found that the optimum ELK1–GO concentration ratio to maximize the interaction between them is 15–40 (Supplementary Fig. 7). Therefore, keeping the ELK1 concentration at 2%, we developed tubes using GO concentrations between 0.05% (corresponding to an ELK1–GO ratio of 40) and 0.15% (corresponding to an ELK1–GO ratio of 15), and, as expected, qualitatively found that the best-defined and most robust tubes were made within this range (Fig. 3b). To quantify and identify the best ELK1–GO combination, we used an established nanotensile test^38^ on tubes made of 2% ELK1 and increasing concentrations of GO (0.05, 0.10, and 0.15%) (Supplementary Section 14). As expected, the strength, the strain at break, and the toughness modulus increased on tubes formed with increasing concentrations of GO (Fig. 3c, Table). However, based on a Weibull statistical distribution, the results revealed that the elastic modulus was highest on tubes fabricated with 0.10% GO (212.90–247.15 kPa) compared to 0.05% (128.78–147.37 kPa) and 0.15% (159.57–208.16 kPa). This result is also visible from the stress–strain curves of the ELK1–GO (Fig. 3c, graph), where the samples made with 0.1% GO show a steeper slope, meaning that the material is stiffer.

In order to further investigate the role of electrostatic interactions, we formed tubes with ELR and GO solutions at varying pHs and again found that more robust membranes formed when the charge difference between both components was marginal (Fig. 3d, e). These results suggest that optimum co-assembly does not solely depend on strong electrostatic forces but rather on a synergistic effect from different factors that we speculate to be electrostatic and hydrophobic forces, H-bonding, and 3D conformation. To confirm this premise, we first synthesized a single repeat of each individual ELR block. Using CD and MD simulations, we verified that these shorter molecules did not exhibit a *Tt* but have similar secondary structure with large amounts of random coil in aqueous environments (Supplementary Fig. 8). Upon mixing with GO, all three single repeat peptides exhibited similar levels of interaction as evidenced by calculation of the binding constant based on fluorescence emission titration (Supplementary Fig. 9). To further dissect the nature of the initial ELK1–GO interactions, we performed MD simulations at 30 °C and found that H-bonding between the ELK1 and GO plays a role and that these interactions can come from both the linear side chain of lysine and the backbone of the peptide (Supplementary Section 18). However, in addition to these ELK1–GO molecular interactions, we hypothesize that the 3D conformation of the full-length ELK1 protein and its ability to cooperatively interact with the GO lamellae play a key role in the formation of the system. To test this hypothesis, and taking advantage of the ELR’s capacity to change its conformation at different temperatures (Fig. 3f, graph, Supplementary Figs. 10 and 12), we assembled tubes using GO and ELK1 (2 wt%) at either below (4 °C), above (45 °C), or the ELK1’s *Tt* (30 °C) (Fig. 3f). While tubes formed at all temperatures, they were more robust and exhibited better-defined multilayers (Supplementary Fig. 11e) and tubular geometry (Fig. 3f, images) at 30 °C, suggesting stronger interactions at this temperature. This enhanced interaction was also investigate by DLS, which revealed the presence of larger ELK1–GO aggregates at 30 °C compared to 4 °C and 45 °C (Fig. 3g) and further confirm that the 3D conformation of ELK1 at the different temperatures plays a key role in its interaction with the GO lamellae, which would in turn affect the diffusion–reaction mechanism and consequently determine the properties of the resulting ELK1–GO tubes (Fig. 3f, images).

### Underlying molecular mechanism of assembly: ELK1–GO aggregates

**Fig. 4:**
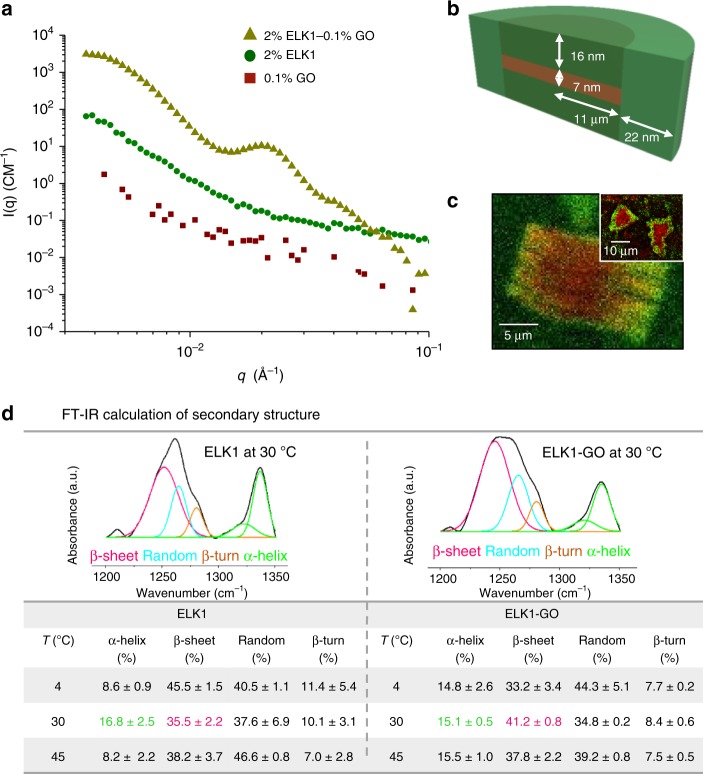
Supramolecular assembly of the ELK1–GO system. **a** Small-angle neutron scattering (SANS) patterns demonstrating a resulting uniform microstructure formed when co-assembling ELK1–GO. Between the middle-*q* region (ca 0.007–0.04 Å^−1^), the ELK1–GO structure (yellow triangle) exhibits a characteristic scattering peak associated with pure ELK1 (green square) and GO (red circle) at 30 °C, confirming the formation of a new order structure different from the individual components. **b** The classical core–shell–bicelle–elliptical model that was fitted to the ELK1–GO microstructure as measured by SANS at 30 °C (green: ELK1, brown: GO). **c** Confocal microscopy (green: ELK1, red: GO) corroborating the interaction between the ELK1 and the GO lamellae (inset depicts the top view of the ELK1–GO structure). **d** FT-IR calculation of secondary structure depicting the change and transition of conformation of the ELK1 molecule before and after binding with GO. At 30 °C and before binding with GO, ELK1 exhibits higher α-helix than at 4 and 45 °C, which is maintained after binding with GO and is complemented with an increase in β-sheet.

To shed light on this enhanced ELK1–GO interaction at 30 °C, we used SANS and found that, as expected, ELK1 exhibited an expanded conformation at 4 °C and a collapsed aggregated conformation with a 74 nm radius of gyration of the core region at 45 °C (Supplementary Fig. 10b). Furthermore, at 30 °C, the molecule acquired a conformation that combined both an expanded structure and a collapsed aggregate core, consisting of a 60 nm radius of gyration of the core region surrounded by a larger 500 nm radius corona of expanded structures (Supplementary Fig. 10b). These different conformations were confirmed by cryo-transmission electron microscopy (cryo-TEM) (Supplementary Fig. 13). On the other hand, GO sheets are known to stack and form lamellae^32^ in aqueous environments. We hypothesized that the disordered nature of ELK1 would facilitate its interaction with the supramolecular framework provided by the GO lamella. To test this hypothesis, we used SANS to investigate the size and shape of the ELK1–GO aggregates upon co-assembly (Fig. 4a, b, and Supplementary Fig. 11a). We found that the scattering profile for the ELK1–GO aggregate formed at 30 °C is better fitted with a classical core–shell–bicelle–elliptical model^39^ with a core measuring 7 nm in length, a thick_rim of 22 nm, and a thick_face of 16 nm (Supplementary Fig. 11d, e). According to this model, the core is formed by GO and the shell by ELK1. This core–shell conformation was confirmed by confocal microscopy (Fig. 4c). On the other hand, at 4 and 45 °C, the ELK1–GO aggregates acquire longer cores and thinner shells (Supplementary Fig. 11d, e), which suggests that at these temperatures the GO lamellae are less infiltrated by ELK1 molecules. In contrast, at 30 °C, the shorter core of the ELK1–GO aggregates indicates that the GO lamellae are more infiltrated by and likely interacting more with the ELK1 (Supplementary Fig. 11d, e).

### Underlying molecular mechanism of assembly: disorder-to-order transitions to enhance integration

It is well-known that proteins rich in disordered regions change their conformation upon binding to other molecules or surfaces^40^. We hypothesize that the enhanced infiltration by ELK1 within the GO lamellae at 30 °C is associated to the disordered nature of the ELK1 and its potential to acquire different secondary structures upon interaction with other molecules. We first used FT-IR amide III spectra to conduct a quantitative analysis of the ELK1’s secondary structure (Fig. 4d)^20,41^. At 30 °C and prior to co-assembly, ELK1 exhibits a high degree of random coil but also higher amounts of α-helix and lower amounts of β-sheet compared to 4 and 45 °C (Fig. 4d). Interestingly, upon binding with GO at 30 °C, ELK1 maintains its α-helix and increases in β-sheet (Fig. 4d). These results suggest that as ELK1 molecules diffuse through the GO lamellae (Fig. 4a–c) at 30 °C, they bind to and interact with GO maintaining their α-helix but increasing their levels of β-sheet (Fig. 4d and Supplementary Figs. 10 and 11). It is known that higher levels of β-sheet conformation generate denser aggregates^42^ and that α-helix proteins lose entropy and increase the system’s stability by aggregating their helices^43^. Therefore, it is possible that these secondary structures enhance the stability of the ELK1–GO complex and consequently lead to a better-defined diffusion barrier at the beginning of the co-assembly process, which is known to have an effect on interfacial membrane assembly^44^. To confirm this, we attempted to form tubular structures using the GO-S, which instead lead to a gel-like structure, suggesting the formation of a loser and more permeable diffusion barrier (Supplementary Fig. 1b).

### Biological validation

**Fig. 5:**
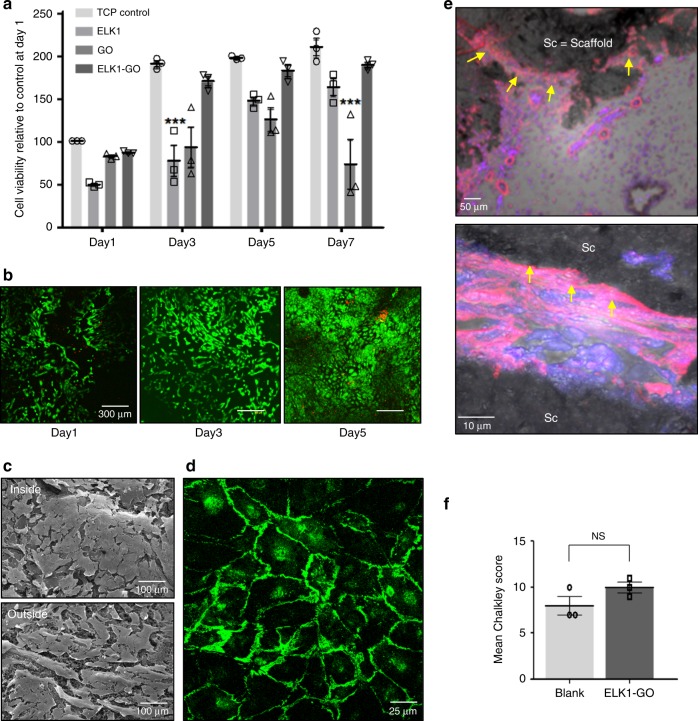
In vitro biocompatibility and bioactivity of the ELK1–GO membrane. **a** The applicability of the material was assessed by an MTS assay to test cell viability and proliferation of hUVECS on both sides of the ELK1–GO membrane. The results revealed that cell viability and proliferation on ELK1–GO materials are at least similar to those of cells growing on tissue culture plastic (TCP) for 7 days. Error bars represent ±s.d. for *n* = 3. *p < 0.05. Two-way ANOVA. **b** Live (green)/dead (red) assay confirmed the proliferation of hUVECs. **c** Scanning electron micrographs demonstrate the formation of an integral endothelial layer on both sides of the ELK1–GO membrane. **d** VE–cadherin (CD144) was labeled to observe the organization of the intercellular junctions and revealed that cells exhibited strong intercellular junction staining, also suggesting the formation of an integral endothelial layer on the ELK1–GO membrane. **e** Histological sections of the ELK1–GO tube implants within a chick chorioallantoic membrane (CAM) model for 7 days highlighting alpha smooth muscle actin (α-SMA, pink), and cell nuclei (blue). The results revealed endothelial cells forming capillary-like structures surrounding the ELK1–GO tubes (yellow arrows). **f** Chalkley count analysis showing a slightly higher level of angiogenesis on tube-containing samples compared to control (blank model) samples. ±s.d. for *n* = 3. *p < 0.05. One-way ANOVA. NS no significance.

The versatility and robustness of the ELK1–GO system offers an exciting possibility to develop complex and functional biohybrid devices with a high level of biological relevance by supramolecular processing. This potential was assessed by suspending human umbilical vascular endothelial cells (hUVECs) within the ELK1 solution prior to co-assembly and growing the tubes described here. Fluorescence microscopy revealed that cells were present both within the assembled ELK1–GO membrane as well as inside the lumen of the corresponding tubes right after co-assembly (Fig. 2g), which is likely a result of cells being either trapped within or adhered to the assembling membrane. Cells were observed to spread and grow for at least 7 days both within the membrane and on the lumen of the tubular structures, indicating that the material is able to support cell survival and growth. To confirm this finding, cell adhesion and proliferation assays were conducted on both sides of ELK1–GO wall of preformed tubes. Remarkably, cells were found to adhere and proliferate at similar levels as those growing on tissue culture plastic (TCP) (Fig. 5a, b), forming a confluent layer on both sides of the membrane (Fig. 5c). To further assess the cell behavior on the tubular structures, VE–cadherin (CD144) was labeled to observe the organization of the intercellular junctions, which are critical for the formation of an intact endothelial monolayer^45^. Confocal images revealed that hUVECs were able to form an integral monolayer on both sides of the ELK1–GO membrane (Fig. 5d).

This notable cell growth and spread on the co-assembled membranes suggests that the hybrid material is cell friendly in vitro. While ELR materials have been shown to support cell growth^46,47^, GO is known to be cytotoxic to endothelial cells in vitro at concentrations higher than 100 ng/mL as a result of plasma membrane damage and oxidative stress^48^. It is important to keep in mind that GO cytotoxicity depends on the inherent properties of the specific GO used^49^. Therefore, we assessed the cytotoxicity of the GO used in this study by conducting experiments using hUVECs in media containing varying GO concentrations and found that our GO is toxic above 0.001% (10 μg/mL) (Supplementary Fig. 14). However, when co-assembling and growing ELK1–GO tubes in the presence of hUVECs, we used GO concentrations that are up to 400× higher than this cytotoxic limit, suggesting that the ELK1–GO complex considerably decreases the cytotoxic level of the GO. This decrease may be due to aggregation of GO sheets^50^ and the localization of the ELK1 on the sharp edges of GO sheets^51^. To investigate potential cytotoxicity as a result of membrane degradation, we exposed tubes to cell culture media for different time points up to 15 days and used extracts from this media to culture hUVECs. In this case, no cytotoxicity was observed for all time points (Supplementary Fig. 15a) compared to hUVECs growing in fresh culture media. Furthermore, by physically damaging ELK1–GO tubes suspended in culture media using strong agitation and exposing hUVECs to this suspension for 72 h, cells exhibited higher viability compared to cells exposed to GO sheets at similar levels of concentration (Supplementary Fig. 15b). To further confirm the biocompatibility of the material, we implanted ELK1–GO tubes directly on an ex vivo preclinical chick chorioallantoic membrane (CAM) model^52^ for 7 days and assessed their cytotoxicity and angiogenesis. Using a Chalkley count analysis, similar angiogenesis was observed on both tube-containing samples and control samples (blank model) (Fig. 5f, Supplementary Fig. 16). Furthermore, immunohistochemistry revealed the presence of capillary-like structures, which in many cases appeared to develop and spread in the vicinity of the ELK1–GO membrane (Fig. 5e). These results are in alignment with previous studies demonstrating the angiogenic potential of GO^48^. However, our approach permits this angiogenic potential while enabling the use of much higher concentrations of GO.

## Discussion

We have demonstrated the possibility to exploit multicomponent self-assembly to hierarchically control the interactions between a disordered protein and GO and grow hybrid materials and devices with a spectrum of new functionalities. The system takes advantage of the inherent properties of both GO and ELRs to trigger a diffusion–reaction process that enables disorder-to-order transitions to facilitate GO–disordered protein interactions, supramolecular integration, and hierarchical assembly. Based on both experimental and simulation evidences, we have described the key steps of the underlying molecular mechanism and established rules to grow the material and easily assemble functional devices. The system exhibits a series of properties that emerge from the synergistic interaction between the two components, including remarkable stability, access to non-equilibrium for substantial periods of time, robustness of assembly, biocompatibility, and bioactivity. We have shown how these properties enable its integration with rapid-prototyping techniques to biofabricate functional microfluidic devices by directed self-assembly, opening new opportunities for engineering more complex and biologically relevant tissue engineered scaffolds, microfluidic systems, or organ-on-a-chip devices. Furthermore, our study addresses a major challenge in materials science by demonstrating the possibility to bridge the gap between supramolecular design and functional and robust biomedical engineering.

## Method

### Chemicals

4-methylbenzhydrylamine (MBHA) rink amide resin and fluorenylmethyloxycarbonyl (Fmoc)-protected amino acids were purchased from Merck Millipore. 1-hydroxybenzotriazole (HOBt) hydrate was purchased from Cambridge Bioscience. N,N′-diisopropylcarbodiimide (DIC), dimethylformamide (DMF), dichloromethane (DCM), piperidine, trifluoroacetic acid (TFA), triisopropylsilane (TIS), diethyl ether, acetonitrile (ACN), acetic anhydride and Kaiser Test Kit were purchased from Sigma-Aldrich and used without further purification. Rhodamine B (≥95%, HPLC grade) and paraformaldehyde (95%) were obtained from Sigma-Aldrich. Two kinds of GO (GO-L with product number-777676; GO-S with product number-763705) were obtained from Sigma-Aldrich. The GOs’ size distribution can be found in Supplementary Fig. 1. Alexa Fluor™ 488 NHS Ester (Succinimidyl Ester) was obtained from Thermo Fisher Scientific.

### Synthesis and characterization of ELRs

ELK0, ELK1, and ELK3 molecules were provided by TP Nanobiotechnology (Valladolid, Spain). Figure 1a shows the sequences, molecular weights, and inverse-phase *Tt* of the ELRs. ELRs were synthesized by *Escherichia coli* recombinant expression system. The sequence and molecular weights of the polymers were verified using amino acid analysis. Sodium dodecyl sulfate–polyacrylamide gel electrophoresis and matrix-assisted laser desorption/ionization-time-of-flight SIMS were used to carry out the ELRs characterization.

### Peptide synthesis and purification

Peptides representative of the single repeat of an individual ELR block were synthesized in a microwave-assisted automated peptide synthesizer (Liberty Blue, CEM) using the standard solid phase peptide synthesis method and Fmoc-protection chemistry. Because the individual blocks are linked together in the ELR molecule through peptide (amide) bonds, synthesized peptides were amidated at the C-terminus and acetylated at N-terminus to resemble the continuation of the amide backbone character and not introducing any additional charges. MBHA rink amide resin was used as the solid support. Amino acid couplings were performed with a mixture of Fmoc-amino acid/HOBt/DIC at a molar ratio of 4:4:4, relative to the resin. Fmoc deprotections were performed with 20% (v/v) piperidine in DMF for 10 min twice. Once all coupling and deprotection reactions were completed, the N-terminal of the peptides were manually capped with 20% acetic anhydride in DMF for 20 min twice. Acetylation reaction was monitored with Kaiser test for free amines. Peptides were then cleaved from the resin with a mixture of TFA/TIS/H_2_O at a volume ratio of 95:2.5:2.5 for 2 h with simultaneous removal of side-chain protecting groups. The cleavage solution was then collected and the excess of TFA removed by rotary evaporation. Cold diethyl ether was added to precipitate the peptide product, which was then collected, washed again with cold diethyl ether and dried under vacuum overnight. Mass of the crude product was analyzed via electrospray ionization mass spectrometry (ESI–MS, Agilent).

Purification of the peptides was performed in an AutoPurification System (Waters) using a preparative reverse-phase C18 column (XBridge, 130 Å, 5 µM, 30 × 150 mm, Waters) and H2O/ACN (0.1% TFA) as mobile phase. Fractions containing the peptides were automatically collected when their exact mass was detected in the SQ Mass Detector (Waters). The collected peptide fractions were lyophilized and stored at −20 °C until further use.

Mass confirmation for all peptides was performed via ESI–MS and their purity analyzed in an Alliance HPLC system (Waters) equipped with an analytical reverse-phase C18 column (XBridge, 130 Å, 3.5 µM, 4.6 × 150 mm, Waters) and monitored at 220 nm.

### Sample preparation (ELRs–GO system)

Aqueous suspension of GO (0.1 wt%, 100 μL) was added to a well of 96-well TCP and aqueous solution of the ELRs (2 wt%, 18 μL) was slowly injected into the suspension of GO. The tip of the pipette was allowed to make contact with the bottom of the well before releasing the ELRs solution vertically at a constant speed. All samples were prepared in MilliQ water.

### Temperature-controlled spectrophotometry

The thermo-responsive behavior of ELK1 at certain concentration (2 wt%) and pH 8 was determined on a temperature-controlled UV–visible spectrophotometer (Agilent Technologies). ELR samples (2 wt%) were prepared in MilliQ water and the pH of the solutions was adjusted with HCl (0.5 M) and NH4OH (1.0 M) prior to heating at 1 °C/min ramping rate. Absorbance of the samples was obtained at *λ* = 350 nm.

### Zeta potential (ζ)

In order to optimize the formation of the ELK1–GO system, the zeta potential of both ELK1 and GO was measured on Zetasizer (Nano-ZS ZEN 3600, Malvern Instruments, UK) at 30 °C under various pH conditions. The concentration of ELK1 and GO used for the measurements is 0.025 and 0.00125 wt%, respectively. The pH values of the two component solutions were adjusted using 0.5 M HCl (at most 3 μL into 1 mL ELK1 or GO solution) and 1.0 M NH4OH (at most 2 μL into 1 mL ELK1 or GO solution) and the samples were equilibrated for 10 min at the set temperature prior to the measurement of zeta potential.

### Dynamic light scattering (DLS)

DLS was performed to measure changes in the particle size of ELK1–GO aggregates at 4 °C (below ELK1’s *Tt*), 30 °C (at the *Tt*), and 45 °C (above the *Tt*). The ELK1 and GO were dissolved in MilliQ water at the concentrations of 0.2 and 0.01% separately. The two solutions were mixed in a 1:1 ratio and the particle sizes were measured using Zetasizer (Nano-ZS ZEN 3600, Malvern Instruments, UK). Samples were equilibrated for 10 min at the desired temperature before measurements.

### Fluorescence emission

Fluorescence emission was measured on LS 55 spectrofluorometer (Perkin Elmer). The aqueous solution of GO (2.5 × 10^−3^ wt%, 1.5 mL) and the solution of various concentrations of ELRs (1.5 mL) were mixed in a 10 mm path length cuvette at 30 °C. The excitation and emission slits were set at 10 nm. The GO was excited at 255 nm and the emission spectra were collected between 300 and 700 nm (200 nm/min). The fluorescence emission intensity was recorded at 518 nm. The data were fitted into the Benesi–Hildebrand equation (1) in order to determine the association/binding constant (Ka) between GO and ELRs.

1 $$1/\Delta I = 1/\Delta I_{\rm{max}} + (1/Ka[C])(1/\Delta I_{\rm{max}})$$

where [*C*] is the concentration of ELRs, ∆*I* = *I* − *I*_min_ and ∆*I*_max_ = *I*_max_ − *I*_min_, where *I*_min_, *I*, and *I*_max_ are the emission intensities of GO considered in the absence of ELRs, at an intermediate ELRs concentration and a concentration of complete saturation, respectively. From the plot of (*I*_max_ − *I*_min_)/(*I* − *I*_min_) against [C]^−1^ for GO, the value of Ka was determined from the slope.

### Circular dichroism (CD)

VT-CD measurements were carried out on Chirascan™ CD Spectrometer (Applied Photophysic Limited, UK) from 10 to 40 °C. The solutions of ELK1 (0.01 wt%) were prepared in MilliQ water and incubated at each temperature for 10 min before measurements. A quartz cuvette with 0.1 cm path length was used for the measurements and CD spectra were obtained by signal integrating 10 scans, from 190 to 260 nm at speed of 50 nm/min. Data were processed by a simple moving average and smoothing method.

### Fourier transform infrared spectroscopy (FT-IR)

FT-IR analysis was conducted on FT-IR spectrometer GX (PerkinElmer^®^, Waltham, MA, USA). A solution of ELK1 (2 wt%) in a mixture of D_2_O and H_2_O (75/25 v/v) and the preformed ELK1–GO membranes prepared in the same solution were properly secured over the IR window before scanning. All samples were incubated and formed at 4, 30, and 45 °C for 10 min before measurements. The program was set to take the average of 160 scans at a resolution of 2 cm^−1^ after subtracting the background and spectra were obtained at wavenumber 4000–600 cm^−1^ with respect to the absorbance for all samples. In order to quantitatively determine the maximum absorption intensity corresponding to various secondary structures of the ELRs (α-helix, β-sheets, β-turns, and random coils) amide III region (1350–1200 cm^−1^) was analyzed using second derivative of a Guassian and Lorentian curve fittings. The second derivative fingerprints for the secondary structures of the ELRs are as follows: 1220–1250 cm^−1^ for β-sheets, 1250–1270 cm^−1^ for random coils, 1270–1295 cm^−1^ for β-turns, 1295–1330 cm^−1^ for α-helix, as previously suggested by Cai et al^41^.

### Scanning electron microscopy (SEM) and wavelength-dispersive spectroscopy (WDS)

The microstructures of ELRs–GO and ELK1–GO membranes cocultured with HUVECs were examined by SEM. ELK1–GO membranes with HUVECs were fixed with 4% paraformaldehyde in MilliQ water for 20 min before dehydration while ELRs-GO membranes were dehydrated directly using increasing concentrations of ethanol (20, 50, 70, 90, 96, and 100%). All samples were subjected to critical point drying (K850, Quorum Technologies, UK) prior imaging. The SEM micrographs were captured on Inspect F50 (FEI Comp, the Netherlands) after sputter-coating with gold (10 nm thick). WDS elemental analyses were performed to study the molecular composition of both the inner and outer surfaces of the ELK1–GO membranes. Quantitative Nitrogen elements (nitrogen exists in ELRs not in GO.) were also analyzed using the Inspect F50 (FEI Comp, the Netherlands). All samples consisting only ELRs or GO were prepared for SEM imaging without a prior cross-linking process.

### Cryo-transmission electron microscopy (Cryo-TEM)

The ELK1 solutions were prepared at 2 wt% in MilliQ water. Grids were vitrified using the Vitrobot MK IV. The Vitrobot chamber was equilibrated to the desired temperature (4, 30, or 45 °C), at 95% relative humidity. Quantifoil R 1.2/1.3 grids were glow discharged in air using the Quorum GloQube^®^ for 60 s, 40 mA. Grid tweezers, grid and pipette tips were preheated to protein aggregate temperature before using. Totally, 3 μL of sample was applied to the grid, blotted for 5 or 6 s with a blot force of 6. Cryo-TEM imaging was performed on a Titan Krios microscope (Thermo Fisher Scientific, US) operating at 300 kV, using a Falcon III direct electron detector.

### Confocal microscopy

The interaction and localization of ELK1 and GO was probed using laser scanning confocal and multiphoton microscopy (TCS SP2, Leica Microsystems, Germany). ELK1 (2 wt%) was dissolved in an aqueous solution of Alexa Fluor™ 488 NHS Ester (10^−6^ wt%) and GO were diluted to  0.1 wt% with an aqueous solution of Rhodamine (10^−6^ wt%). All solutions were incubated for 20 min at 30 °C and protected from light. The tubes were fabricated with 50 μL GO-Rhodamine solution and 10 μL ELK1-Alexa Fluor solution in a 96-well Petri dish as previously described. Images were acquired at laser wavelengths of 488 and 543 nm which correspond to the excitation wavelength of Alexa Fluor and rhodamine, respectively. Images were further processed using ImageJ.

### Small-angle neutron scattering (SANS)

The GO suspension and ELK1 were dissolved in H_2_O/D_2_O (25%/75%) respectively with 0.1 and 2%. SANS measurements were performed on the fixed-geometry, time-of-flight LARMOR diffractometer (ISIS Neutron and Muon Source, Oxfordshire, UK). A white beam of radiation with neutron wavelengths spanning 2.2 to 10 Å was enabled access to *Q* [*Q* = 4*π*sin (*θ*/2)/*λ*] range of 0.004–0.4 Å^−1^ with a fixed-sample detector distance of 4.1 m. Solutions (0.4 mL) of individual components were contained in 1 mm path length UV spectrophotometer grade quartz cuvettes (Hellman) while the composite materials were prepared by mixing equal volume (0.2 mL) of both components in a demountable 1 mm path length cuvettes. The cuvettes were mounted in aluminum holders on top of an enclosed, computer-controlled sample chamber at 30 °C. For the variable temperatures (VTs) experiment (especially those involving ELK1 at 4, 30, and 45 °C), a thermostatted circulating water bath was fitted with the sample chamber. Time taken for each measurement was approximately 30 min. All scattering data were normalized for the sample transmission, the backgrounds was corrected using a quartz cell filled with D_2_O or H_2_O/D_2_O (25%/75%) and the linearity and efficiency of the detector response was corrected using the instrument-specific software.

In the present SANS experiments, we consider that the scattering length density (SLD) of the H_2_O/ D_2_O (25%/75%) is a volume fraction weighted average of the SLDs of the individual components. Given the SLDs for H_2_O and D_2_O are −5.6 × 10^−7^ Å^−1^ and 6.3 × 10^−6^ Å^−1^, we determined the SLD of the H_2_O/D_2_O (25%/75%) is 4.653 × 10^−6^ Å^−1^. The neutron scattering length densities for the GO, H_2_O/D_2_O, and ELK1 are summarized in Supplementary Fig. 11c.

### Cell culture

Human umbilical vein endothelial cells (hUVECs) (Lonza, Isolated in EGM™-2 Media, C2519A) were cultured in EGM™-2 Media (Lonza, CC-3156 and CC-4176). The medium was changed every 3 days until the cells reached 80% confluency. hUVECs between passage 2 and 4 were used for experiments. The tubes were first washed three times with phosphate-buffered saline (PBS) 8 h after assembly and sterilized with UV for 45 min. Then each tube was placed in a well of 48-well cell culture plate with inner or outer side facing up. The EGM™-2 Media (500 μL) containing 50,000 cells was added to each well containing ELK1–GO membranes, coated with ELRs solution (18 μL, 2 wt%), GO (20 μL, 0.1 wt% GO) or on TCP (positive control). The coated wells were incubated for 8 h prior to cell seeding. The cells were incubated at 37 °C and 5% CO_2_ for different time points for all tests (protocol shown below).

### Cell viability and proliferation assay and cytotoxicity assay

The effect of ELK1–GO membranes on hUVECs viability and proliferation using the CellTiter 96® Aqueous One Solution Cell Proliferation Assay (Promega, Southampton, UK). Cells were seeded at a concentration of 50,000 cells/well in 48-well plates. After incubation for 24 h, 1 d, 3 d, 5 d, 7 d, cell culture medium was aspirated and 500 μL of EGM™-2 Media containing 10% MTS reagent was added to each well. Plates were subsequently incubated for 3 h at 37 °C and the absorbance was read at 490 nm using Infinite F50 plate reader (Tecan, Switzerland). Five replicates of each condition were performed with each assay repeated in triplicate. The cell viability was determined as a percentage of control cell viability and proliferation.

A LIVE-DEAD^®^ Cytotoxicity Assay Kit (Invitrogen, USA) was used to measure the viability of hUVECs seeded on the ELK1–GO membranes. Five replicates of each condition were performed with each assay repeated in triplicate. A stock solution containing calcein AM (1 μM) and ethidium homodimer (2 μM) in PBS was prepared according to the assay instructions, and 200 μL of stock solution was added to each well. Fluorescence images were captured on laser scanning confocal and multiphoton microscopy (TCS SP2, Leica Microsystems, Germany). Viable cells were stained green with calcein AM (ex 495 nm, em 530 ± 12.5 nm), while dead cells red with ethidium homodimer (ex 528 nm, em 645 ± 20 nm).

GFP-hUVECs (Fisher scientific, Angio Proteomie GFP-hUVECs, NC0601093, USA) were used to assess the cytotoxicity of GO by culturing >95% confluent GFP-hUVECs in media containing varying GO concentrations (0, 0.001, 0.0025, 0.005, 0.01, 0.25, 0.05, and 0.1%) and cultured for 48 h. Fluorescent images were taken at 1, 8, 24, and 48 h to assess the cytotoxicity by morphology and confluency analysis.

GFP-hUVECs were also used to assess the potential cytotoxicity of ELK1–GO degradation products (extracts). We exposed 1 tube fabricated by 100 µL 0.1% GO and 18 µL 2% ELK1 to 1 mL cell culture media for different periods of time s (1, 3, 6, 9, 12, and 15 days) and used the extracts from this media to culture >95% confluent GFP-hUVECs for 7 days. Fluorescent images were taken at day 1 and day 7 to assess the cytotoxicity by morphology and confluency. To further assess the potential physical degradation of ELK1–GO, physically damaged ELK1–GO tubes using strong agitation were suspended in culture media and exposing >95% confluent GFP-hUVECs to this suspension for 72 h. Fluorescent images were taken to assess the cytotoxicity.

### Immunofluorescence staining

hUVECs on the ELK1–GO membrane were fixed with 4% paraformaldehyde (Sigma, USA), washed and permeabilized with 0.5% Triton X-100 (Sigma, USA), and then rinsed 3 times with PBS. Nonspecific binding sites were blocked by PBS containing 1% bovine serum albumin. The CD144 marker was labeled by incubating the cells at room temperature for 1 h with anti-rabbit monoclonal VE–cadherin primary antibody (1:400, ab33168, Abcam, UK). Cells were then washed and incubated for 50 min at room temperature in Alexa 488 conjugated anti-Rabbit IgG as Secondary Antibody (1:1000, R37116, Invitrogen, USA). The stained ELK1–GO membranes were then transferred to slides and visualized on a laser scanning confocal and multiphoton microscopy (TCS SP2, Leica Microsystems, Germany) utilizing ×10 and ×40 objectives.

### Chick chorioallantoic membrane (CAM) assay

Fertilized chick eggs (Gallus domesticus) were kept in a hatchmaster (Brinsea, UK) incubated at 37.5 °C and humidified with rotation. Twelve (six per group: blank control group and ELK1–GO group) day 1 fertilized eggs were maintained within the hatchmaster. After candling the egg to determine if the egg is fertilized a window was created at day 7 under sterile conditions. A window was created by scoring with a scalpel and an approximately 6 mm square opening created in the outer shell of the egg. The membrane was removed from the underlying CAM vascular membrane. ELK1–GO tube samples were inserted into the window and onto the chorioallantoic membrane. Eggs were transferred to a Hatchmaster incubator and incubated for a duration of 8 days at 37.5 °C 60% humidity without rotation. All procedures were performed in accordance with ethical approval and in accordance with the Animal (Scientific Procedures) Act 1986, UK (Project License number P3E01C456). After 8 days of the CAM culture the implanted samples were harvested.

### Immunohistology staining

Slides were first deparaffinized by washing in two changes of xylene (Sigma, UK) and graded ethanol baths (absolute ethanol, 90%, 70%). Antigen retrieval was performed to unmask the antigenic epitope of the tissue sample by boiling the deparaffinized sections in citrate buffer (Vector laboratories, UK) at pH 6.0. Endogenous peroxidase activity was blocked by incubating sections in 3% H_2_O_2_ solution (Sigma, UK) in PBS at room temperature for 10 min followed by two rinses in PBS. To reduce background staining and any other immunostaining application, the samples were incubated with normal goat serum (5% in PBS, Vector laboratories, UK) to block nonspecific binding sites in a humidified chamber at room temperature for 1 h before staining. After draining the blocking buffer, 100 µL of diluted primary anti-α-SMA antibody (1:500, ab5694, Abcam, UK) was added to the sections on the slides and incubated in a humidified chamber at room temperature for 1 h, after which the slides were washed twice in PBS. Then, 100 µL of ready-to-use biotinylated anti-mouse/rabbit IgG secondary antibody (Ready-to-use, PK-7200, Vector laboratories, UK) was applied to the sections on the slides and incubated in a humidified chamber at room temperature for 30 min with the slides washed in PBS after that. Amplification of antigen was achieved using an Elite^®^ ABC-HRP Kit (PK-7200, Vector Laboratories, UK) and positive staining was visualized by incubating in a peroxidase substrate solution using a DAB Peroxidase (HRP) Substrate Kit (PK-7200, Vector laboratories, UK).

### Analysis of goldner’s trichrome staining by Chalkley count

The Chalkley point-overlap morphometric technique is a relative area estimate method to measure the abundance of microvessels in an immunohistochemical sample. A “Chalkley point array graticule” was used to fit onto the eyepiece of a microscope. This graticule consists of a grid that contain 25 random dots which can be rotated 360°. An observer can overlay these dots over structures that have stained positively with goldner’s trichrome. The rotational position with the most dots that land on positively stained structures is described as the “Chalkley count” and samples have higher counts are considered to contain a greater abundance of blood vessels. A blank histological slide sample and three ELK1–GO histological slide samples were scoring by this technique.

### Co-assembly of ELK1–GO–hUVECs

EGM™-2 Media containing hUVECs (10^5^ cells/ml) was used to dissolved the ELK1 (2 wt%). The ELK1–hUVECs media (10 μL) was added into GO (50 μL, 0.4 wt%) solution to make a tube as previously described. All these co-assembled ELK1–GO–hUVECs tubes were incubated at 37 °C, 5% CO_2_ for 24 h, 1 d, 3 d, 5 d prior to LIVE-DEAD^®^ cytotoxicity Assay and SEM procedures as described previously.

### 3-D printing of ELK1-GO materials

A PAM2 system (Centro Piaggio, Pisa University, Italy) was applied for the 3-D printing of ELK1–GO materials. Blue food dye (5 μL) was added into aqueous solution of ELK1 (2 mL, 2 wt%) to make the printing procedure visible. For fabricating the different shapes of structures and the 60 μm diameter small tube, a 65 μm diameter glass tube tip was used as nozzle to release the solution of the ELK1 and the dye under 4 kPa pressure at a range of speed between 10 and 18 mm/s. The printing nozzle is merged in a container with 0.1% GO MilliQ water solution. All the 3-D pathway was controlled by the Repetier software. A peristaltic pump was used to perfuse 1 v/v green food dye in MilliQ water. For the vertical tube, the perfusion speed is from 4.7 to 8.3 mL/min. For other structures, the perfusion speed was 2 mL/min.

### Statistical analysis

GraphPad Prism 5 was applied for data analysis. Studentʼs *t-*test statistical analysis was applied for all the measured data.

### Reporting summary

Further information on research design is available in the Nature Research Reporting Summary linked to this article.

## Supplementary information

Supplementary Information

Description of Additional Supplementary Files

ELK1-GO system forming closed sac and opened tube.

ELK1-GO system grown into vertical tubes.

Making of ELK1-GO tube bridging two surfaces: horizontal growth.

3D printing of the ELK1-GO system.

Fluidic device made by printing/self-assembling the ELK1-GO system

bifurcation of a fluidic device made by printing/self-assembling the ELK1-GO system

a tubular fluidic device made by self-assembling the ELK1-GO system showing the flow of water with green food dye and manually removing the flow.

Tubular fluidic device made by self-assembling the ELK1-GO system showing the flow of water with green food dye and self-circulate for 24h without any damage.

Reporting Summary

## Data Availability

The data that support the findings of this study are available from the authors on reasonable request, see author contributions for specific data sets.
