# Supplementary material for: Disordered protein-graphene oxide co-assembly and supramolecular biofabrication of functional fluidic devices
Source: Nat Commun. 2020 Mar 4;11:1182. doi: 10.1038/s41467-020-14716-z (PMC7055247; doi:10.1038/s41467-020-14716-z)
Supplement: Supplementary file 1 — Supplementary Information [file 41467_2020_14716_MOESM1_ESM.pdf]

Supplementary Information

Disordered protein-graphene oxide co-assembly and supramolecular biofabrication of functional fluidic devices

Wu et al

Supplementary Information Contents:

- **Supplementary Section 1:** Lateral size distribution of the two GOs used with different dimensions and structure of ELK1-GO-S
- **Supplementary Section 2:** Single repeat of individual ELR block peptides information
- **Supplementary Section 3:** Gradient of ELK1 from inside to outside of tube
- **Supplementary Section 4:** Interaction between GO and ELRs with different levels of hydrophobicity and charge
- **Supplementary Section 5:** Optimal interaction concentration ratio between ELK1 and GO
- **Supplementary Section 6:** Secondary structure calculation of single repeat of individual ELR block peptides by Circular Dichroism (CD)
- **Supplementary Section 7:** Interaction between GO and single repeat of individual ELR block peptides
- **Supplementary Section 8:** Small-angle neutron scattering (SANS) measurements
- **Supplementary Section 9:** Secondary structure calculation of ELRs Circular Dichroism (CD)
- **Supplementary Section 10:** Cryo-transmission electron microscopy (TEM) image of ELK1
- **Supplementary Section 11:** Cytotoxicity measurement of GO concentration
- **Supplementary Section 12:** Cytotoxicity measurement of ELK1-GO degradation
- **Supplementary Section 13:** Chalkley Score
- **Supplementary Section 14:** Statistical analysis of nanotensile mechanical tests
- **Supplementary Section 15:** Zeta potential (ζ) of ELK1 and GO
- **Supplementary Section 16:** Ion strength affection of ELK1-GO system
- **Supplementary Section 17:** Polydispersity index (PDI) of Dynamic Light Scattering (DLS)
- **Supplementary Section 18:** Computer modelling
- **References**

Supplementary Section 1. Lateral size distribution of the two GOs used with different dimensions and structure of ELK1-GO-S

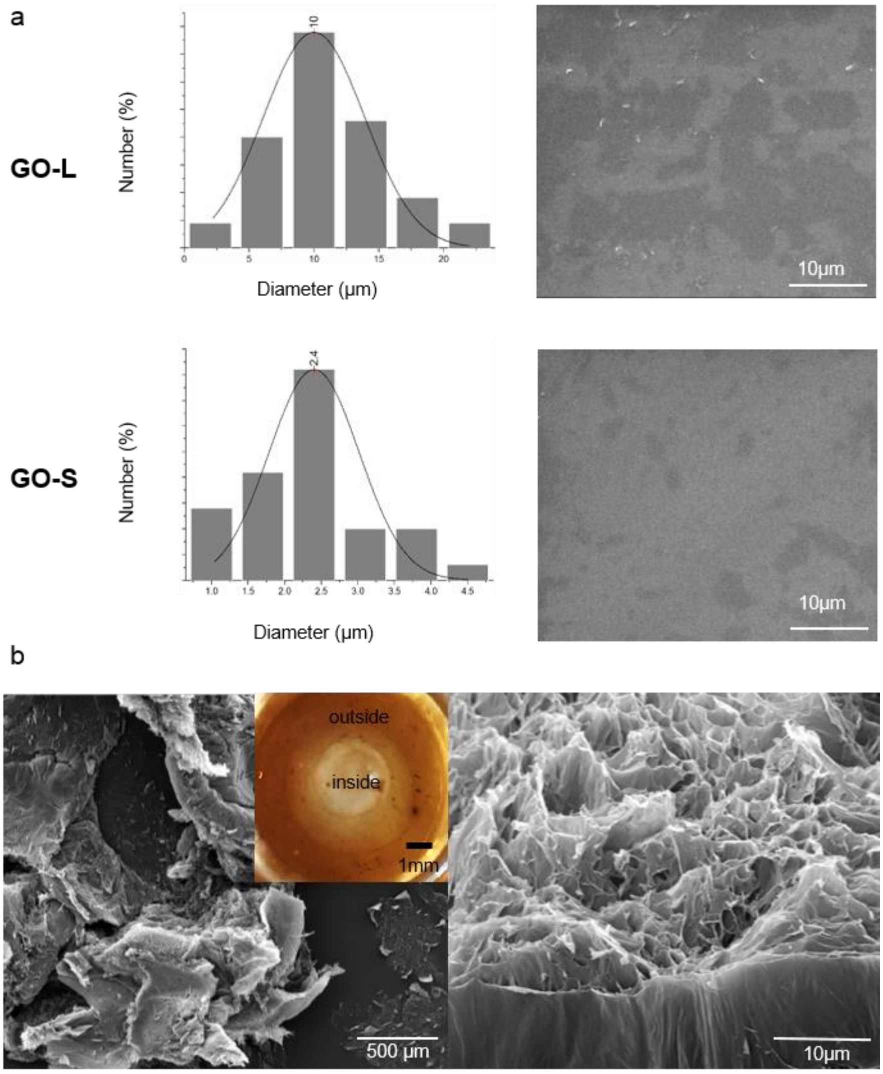

Supplementary Figure 1 a. Lateral size distribution of GO-L (10.5 ± 4.5 μm) and GO-S (2.3 ± 0.9 μm). **b.** SEM images of dried xerogel of ELK1-GO-S. Inset: Optical image of the gel of ELK1-GO-S.

Supplementary Section 2. One repeat single block peptides information

a

ELR peptides	Sequence	Molecular weight (Da)
ELK0-1	CH3CONH-(VPGIG-VPGIG-VPGIG-VPGIG-VPGIG)-CONH2	2176.61
ELK1-1	CH3CONH-(VPGIG-VPGIG-VPGKG-VPGIG-VPGIG)-CONH2	2191.63
ELK3-1	CH3CONH-(VPGIG-VPGKG-VPGKG-VPGKG-VPGIG)-CONH2	2221.63

b

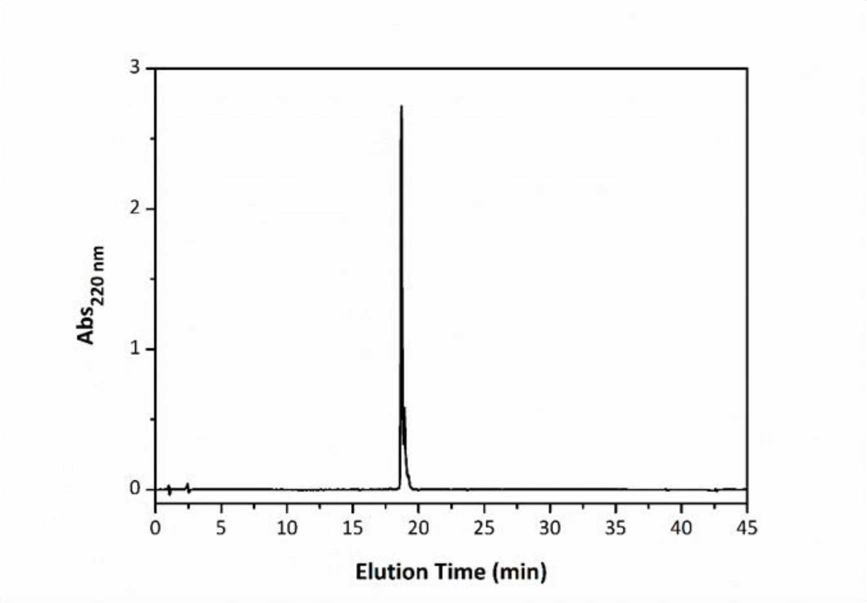

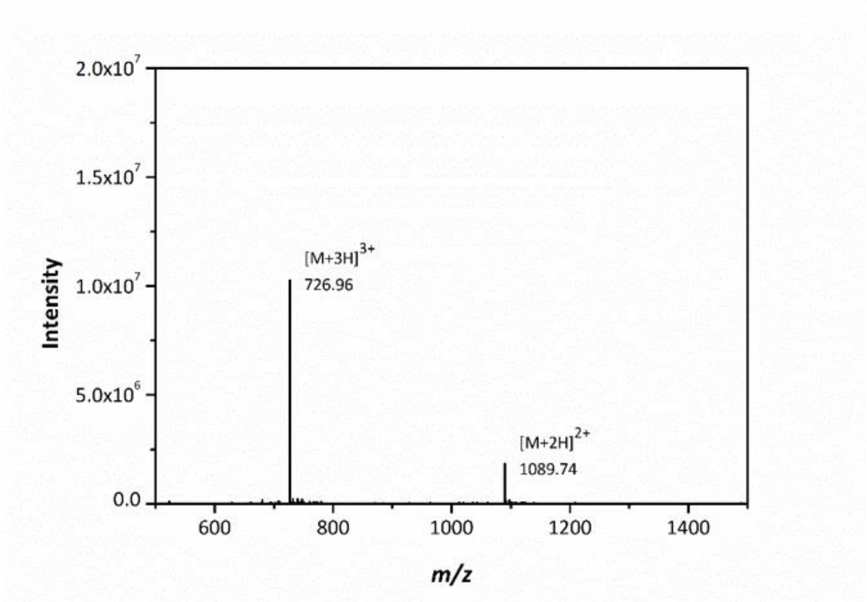

Supplementary Figure 2. Molecular information of ELR peptides. 2b. Analytical RP-HPLC chromatogram (top) of ELK0-1 under the gradient of 98% to 0% H_2O (2% to 100% ACN) with 0.1% TFA from 5 to 35 min showing high purity. ESI-MS spectrum (bottom) of ELK0-1 displaying the expected molecular mass ($C_{102}H_{17}0N_{26}O_{26}$, Mw: 2176.61g/mol)

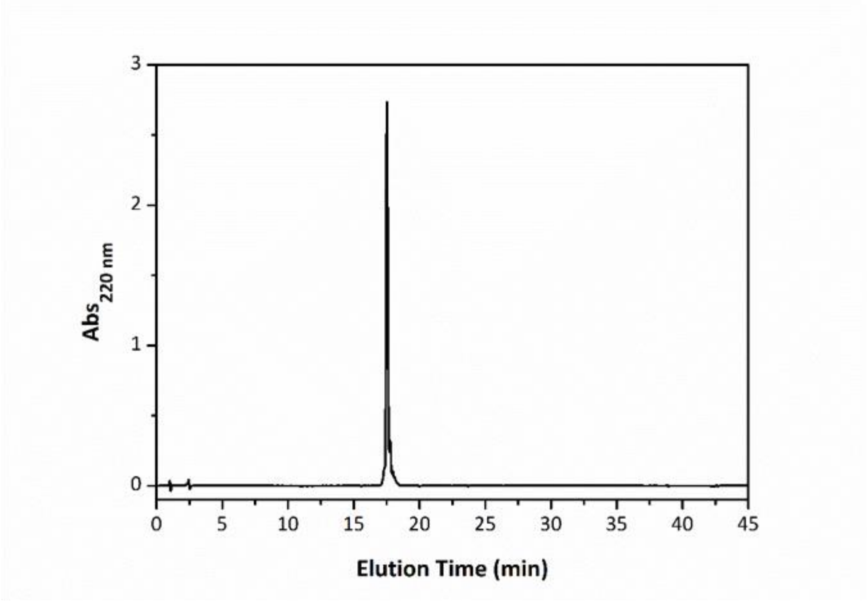

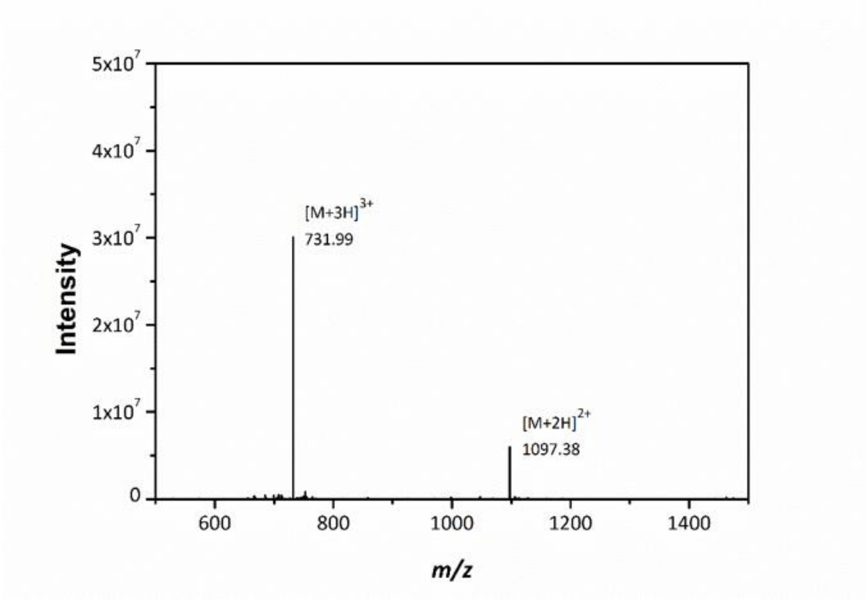

Supplementary Figure 3. Analytical RP-HPLC chromatogram (top) of ELK1-1 under the gradient of 98% to 0% H_2O (2% to 100% ACN) with 0.1% TFA from 5 to 35 min showing high purity. ESI-MS

spectrum (bottom) of ELK1-1 showing the expected molecular mass ($C_{102}H_{171}N_{27}O_{2}6$, Mw: 2191.63 g/mol).

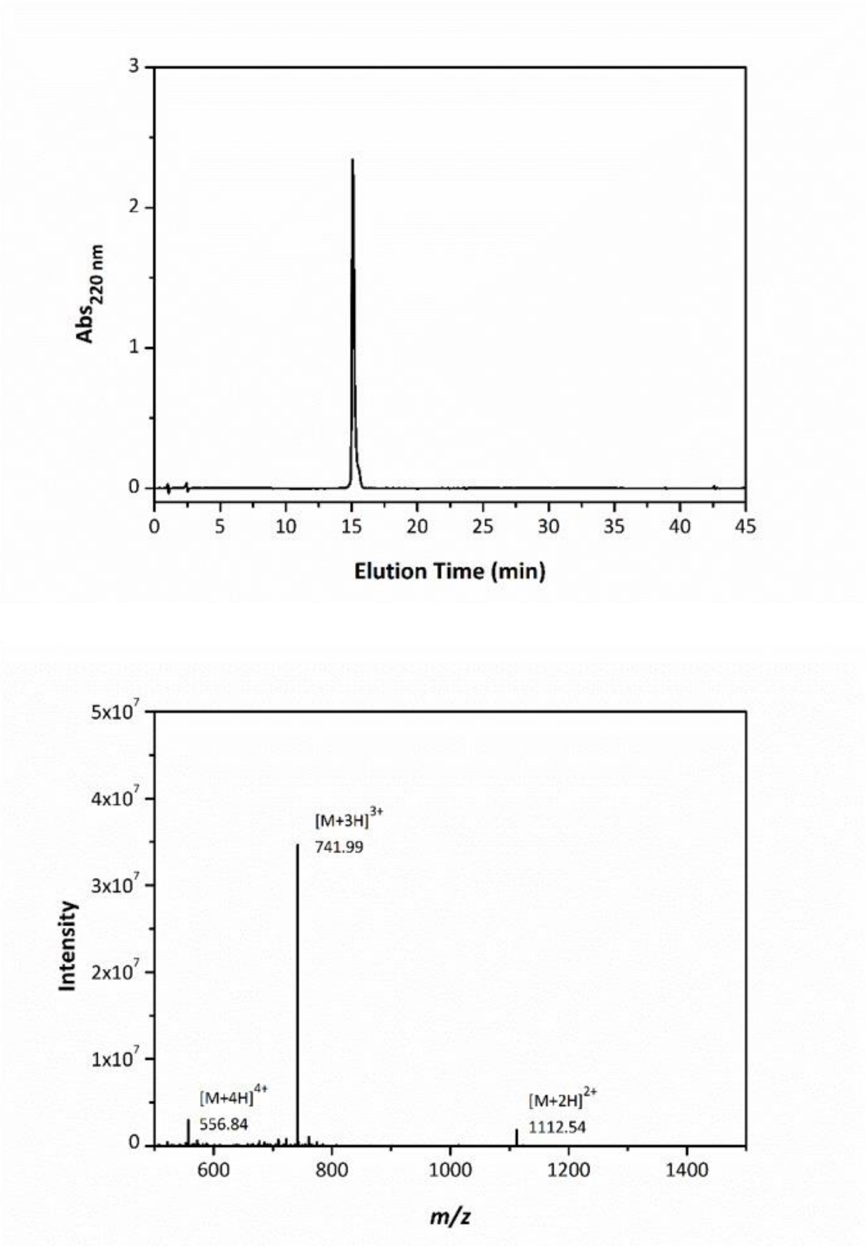

Supplementary Figure 4. Analytical RP-HPLC chromatogram (top) of ELK3-1 under the gradient of 98% to 0% H_2O (2% to 100% ACN) with 0.1% TFA from 5 to 35 min showing high purity. ESI-MS spectrum (bottom) of ELK3-1 showing the expected molecular mass ($C_{102}H_{173}N_{29}O_{26}$, Mw: 2221.63 g/mol).

Supplementary Section 3. Gradient of ELK1 from the tube inside to the outside

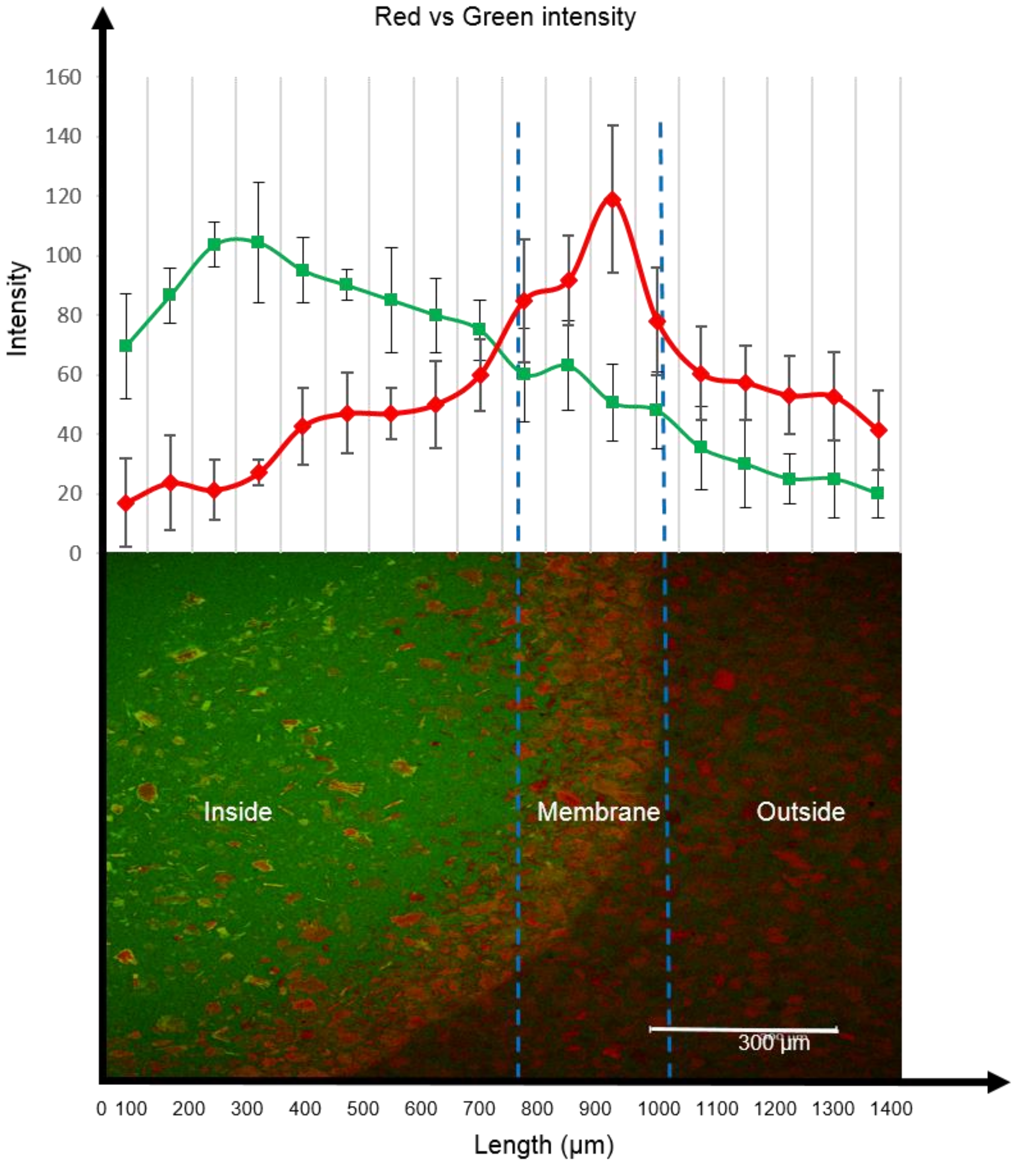

Supplementary Figure 5. Fluorescence intensity calculation of membrane (Green: ELK1. Red: GO) shows the gradual decrease of ELK1 from inside to outside.

Supplementary Section 4. Interaction between GO and ELRs with different levels of hydrophobicity and charge

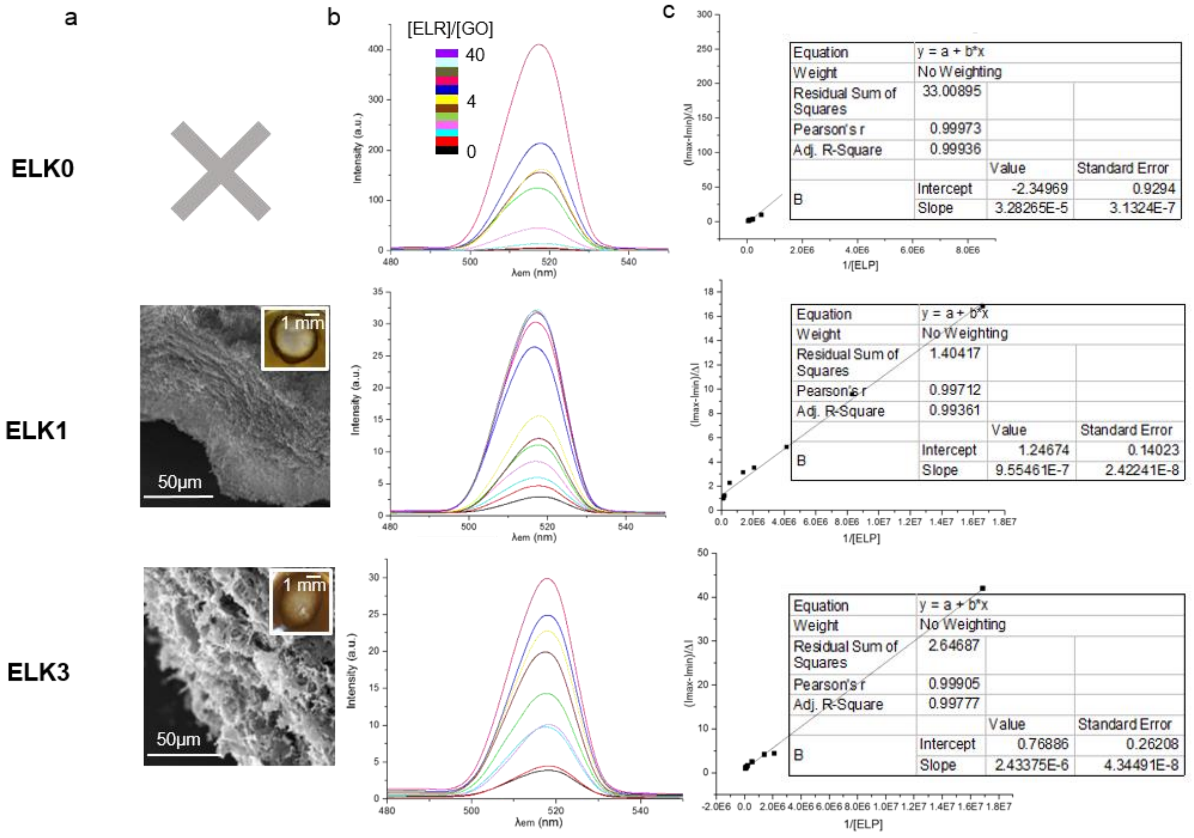

Supplementary Figure 6 a. (ELK0) No stable structure was obtained with ELK0-GO. (**ELK1**) Robust tube with a hierarchical architecture was obtained with ELK1-GO. (**ELK3**) A tube was obtained with ELK3-GO with a rough multilayer membrane at the interface. **4b**. Characterization of GO binding with ELRs by fluorescence emission titration of a fixed concentration of GO ($2.5x10^{-3}$ wt%) and increasing concentrations of ELRs. **4c**. Associated binding constants (Ka) were calculate by Benesi-Hildebrand equation based on the titration results.

Supplementary Section 5. Optimal interaction concentration ratio between ELK1 and GO

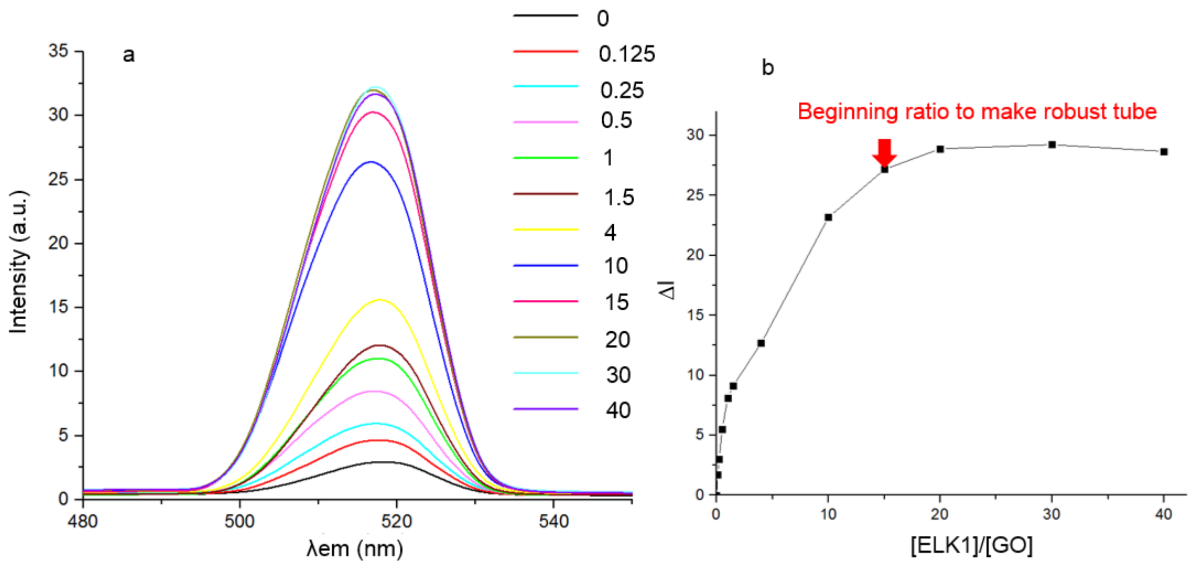

Supplementary Figure 7 a. Characterization of GO binding with ELK1 by fluorescence emission titration of a fixed concentration of GO (2.5x10^{-3} wt%) and increasing concentrations of ELRs. The fluorescent intensity of the peak at 518 nm will continue increasing with increments of the ELK1 concentration for more interaction between them until GO is saturated. **5b.** A graph was made between the [ELK1]/[GO] and peak intensity at 518 nm showing an inflection point at [ELK1]/[GO] equal to 15, the curve reach a plateau above 15.

Supplementary Section 6. Secondary structure measurement of single repeat ELR block peptides by circular dichroism (CD)

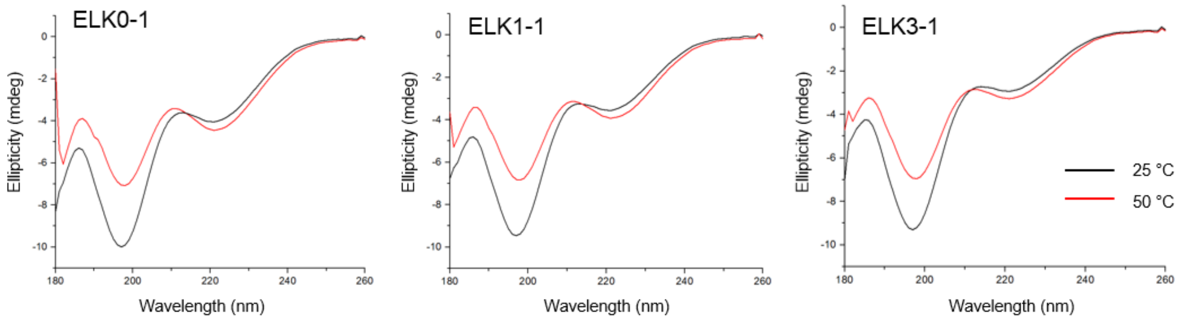

Supplementary Figure 8. CD spectra of the single repeat ELR block peptides show similar little increase of β-turn from 25 °C to 50 °C, while a large percent of random structure was kept in three peptides.

Supplementary Section 7. Interaction between GO and single repeat ELR block peptides

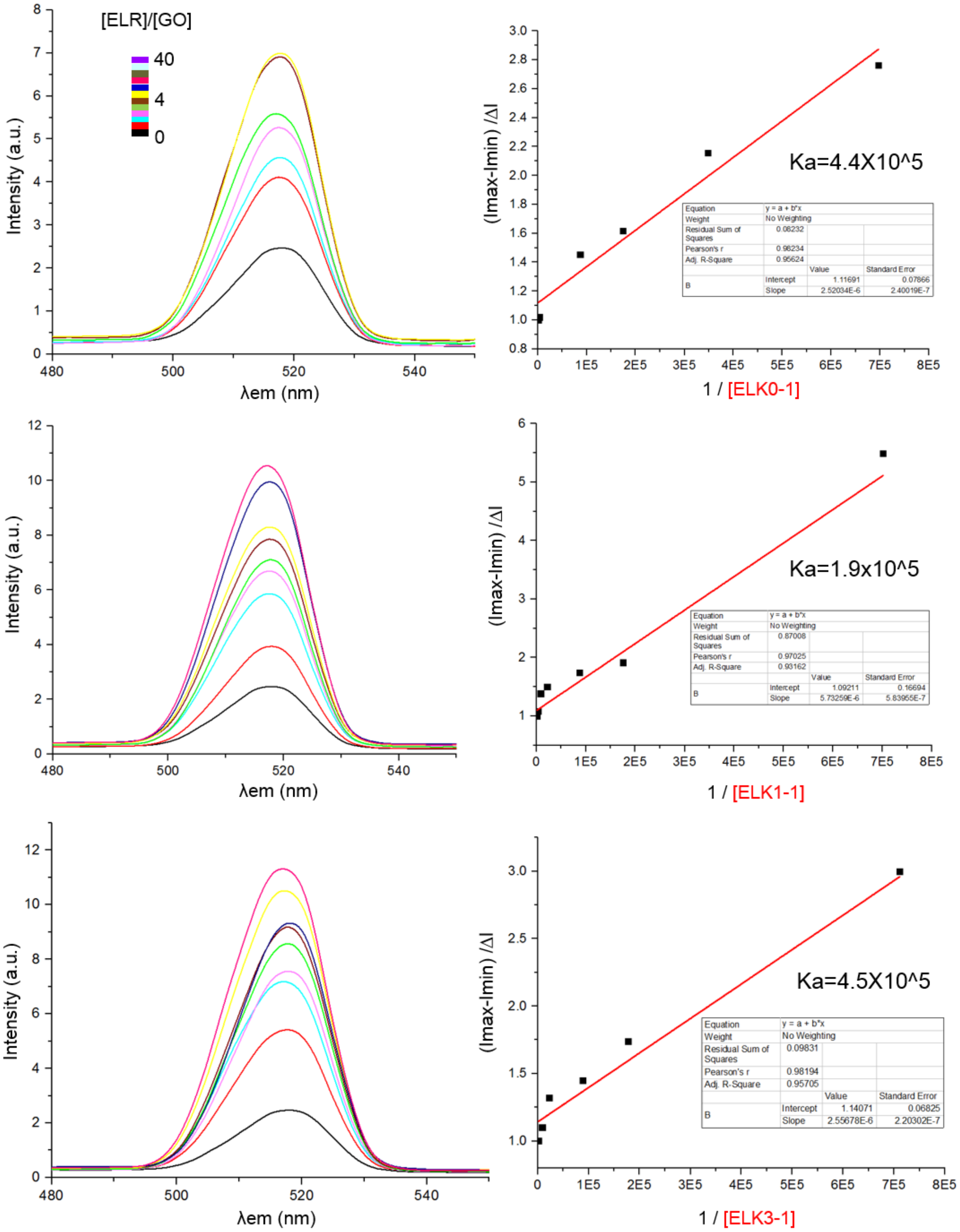

Supplementary Figure 9. Characterization of GO binding with ELRs by fluorescence emission titration of a fixed concentration of GO ($2.5x10^{-3}$ wt%) and increasing concentrations of single repeat ELR block peptides (left). Associated binding constants (Ka) were calculate by Benesi-Hildebrand equation based on the titration results (right).

Supplementary Section 8. Small-angle neutron scattering (SANS) measurements

a

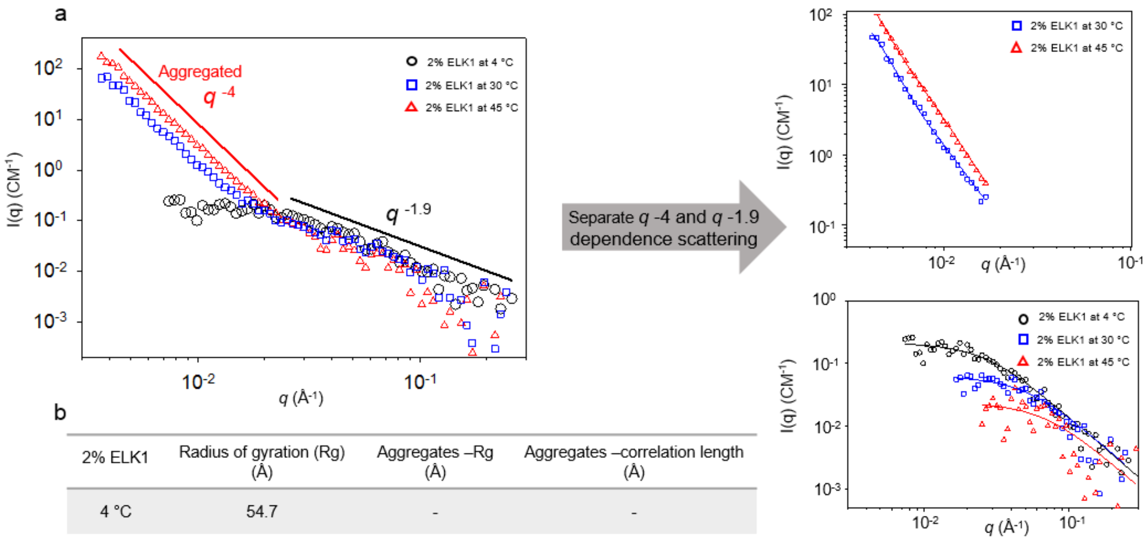

b

2% ELK1	Radius of gyration (Rg) (Å)	Aggregates –Rg (Å)	Aggregates –correlation length (Å)
4 °C	54.7	-	-
30 °C	28.7	599.2	5769.8
45 °C	21.5	741.6	338.9

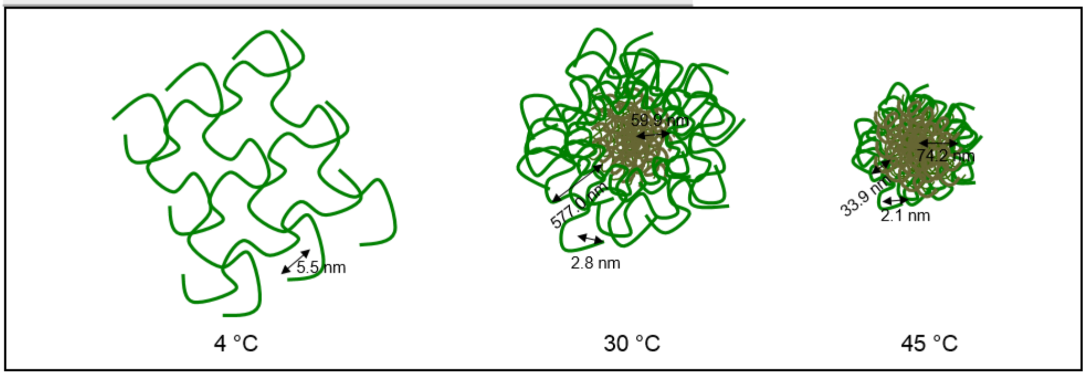

Supplementary Figure 10. SANS results (shapes), fittings (lines), and diagrams for ELK1 at different temperatures (4, 30 and 45 °C). **a (left).** Pronounced q^{-4} dependence scattering appear at 30 °C and 45 °C at low q (*ca* 0.0035-0.02 Å^{-1}), which demonstrates the scattering is from aggregates with smooth surface. At high q range, the I vs q curves at all temperatures follow a new power law of approximately $q^{-1.9}$, which indicates the scattering is from gaussian chains in a dilute environment. **a (right).** Furthermore, we separated the q^{-4} and $q^{-1.9}$ dependence scattering. A gel-fit model was used to fit q^{-4} porod scattering to obtain the Rg and correlation length of the aggregate structure at 30 °C (blue square) and 45 °C (red triangle) **(top)** and a poly-gauss-coil model was used to fit the ELK1 $q^{-1.9}$ dependence scattering to obtain the Rg of the gaussian chains at different temperatures**(bottom). b.** Parameters and diagram were given by the best fitting of poly-gauss-coil/ get-fit model for ELK1 at different temperatures (Green: ELK1 liner structure. Brown: ELK1 aggregates).

a

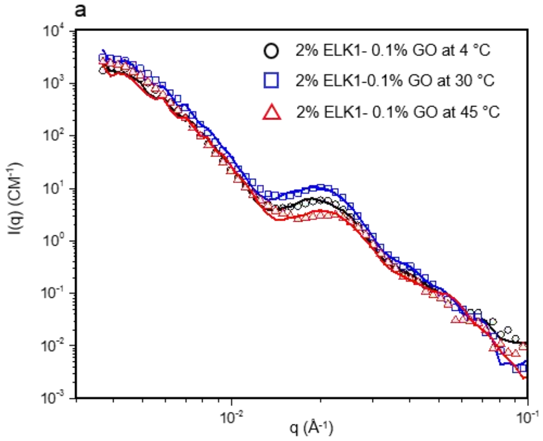

b

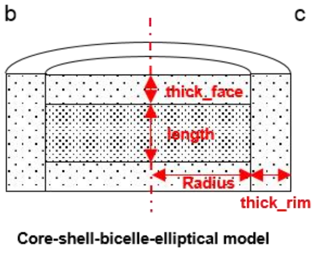

c

Material	SLD (10^{14} Å^{-2})
$H_2O(0.25)D_2O(0.75)$	6.53
GO	~6
ELK1	1.65

d

2% ELK1-0.1% GO	Thick_rim (Å)	Thick_face (Å)	Length (Å)	Radius (Å)
4°C	79.0	121.3	154.9	109460
30°C	216.2	163.9	69.7	110860
45°C	59.9	131.4	130.6	111490

e

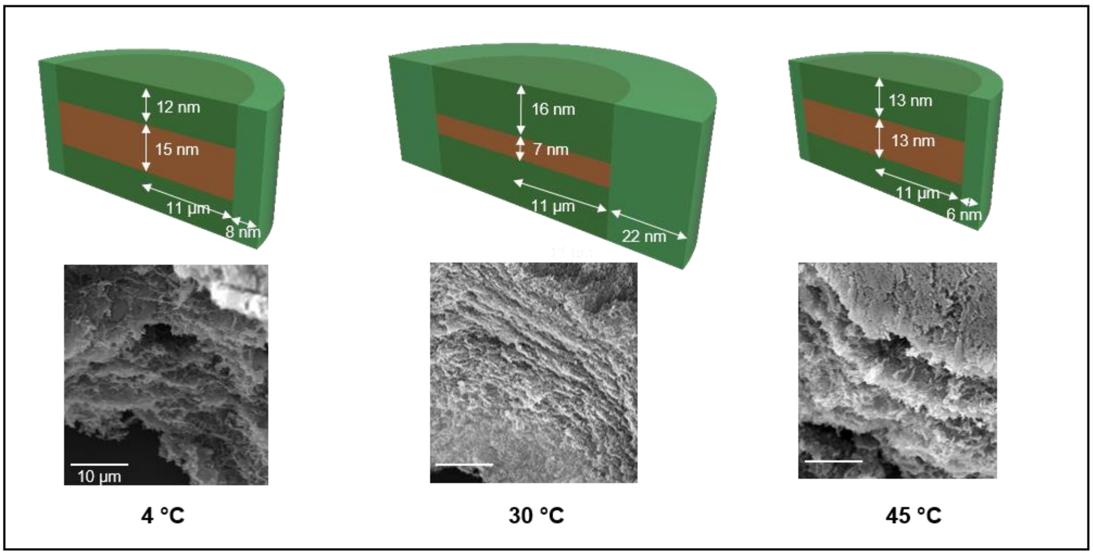

Supplementary Figure 11. SANS results (shapes), fittings (lines), fitting parameters, and diagrams for ELK1-GO at different temperatures. **a.** SANS measurement and fitted curves of the hybrid ELK1-GO structure at 4, 30 and 45 °C. Based on physical understanding, confocal microscopy results and using the SasView[1] software program, all of these three curves were fitted well by a core-shell-bicelle-elliptical model, as shown in **b.** The length and thick-rim of the core-shell-bicelle-elliptical model represent the length of the GO and thick_rim of the ELK1 in the ELK1-GO ordered structure, for which the scattering length density[2] is summarised in **c. d.** Critical parameters of the best fitting of the ELK1-GO structure at different temperatures using the core-shell-bicelle-elliptical model. **e.** Diagrams of the best fitting core-shell-bicelle-elliptical model for the ELK1-GO structures (green: ELK1, brown: GO) at different temperatures detailing the respective sizes and indicating more infiltration of ELK1 within GO at 30 °C

compared to 4 and 45 °C, evidenced by the shorter GO core. This result is supported by the thinner and more-defined layers of the ELK1-GO membrane cross-section at 30 °C as evidenced by SEM.

Supplementary Section 9. Secondary structure measurement of ELRs by CD

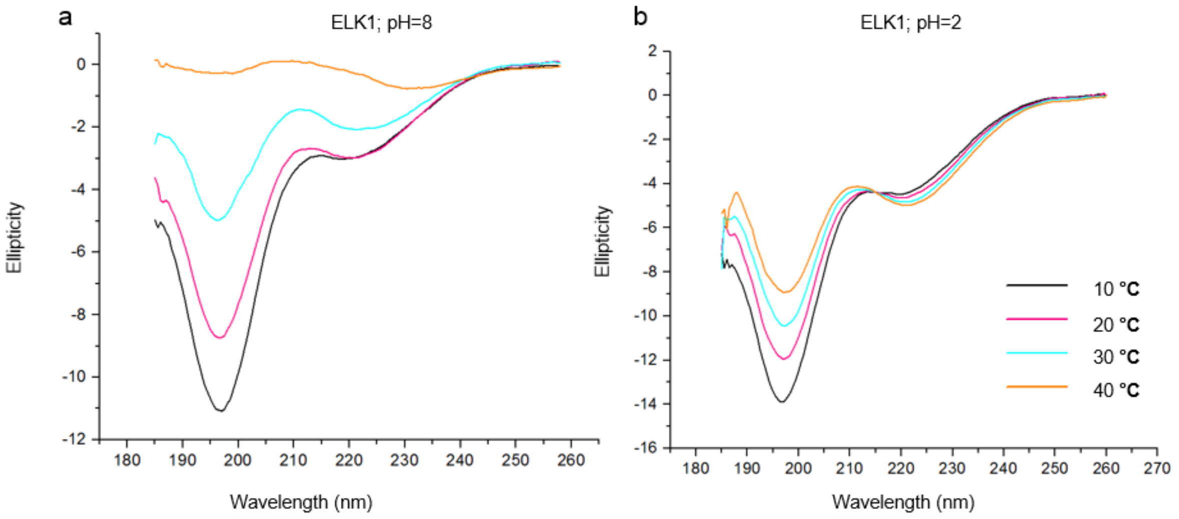

Supplementary Figure 12 a. CD spectra of 0.01% ELK1 in MilliQ water at pH 8 shows an increasing α-helix pattern. 9b. CD spectra of 0.01% ELK1 in MilliQ water at pH 2 shows an increasing β-turn pattern.

Supplementary Section 10. Cryo-transmission electron microscopy (TEM) imaging of ELK1 below, at and above Tt

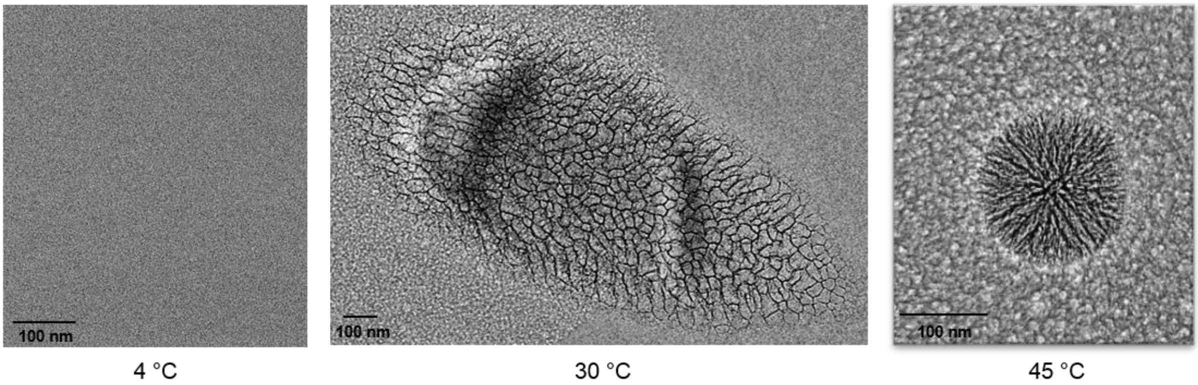

Supplementary Figure 13. Cryo-TEM images of aqueous solution of ELK1 (2%) at 4°C, 30 °C, and 45 °C. At 4 °C, there are no visible protein aggregates. At 30 °C show the ELK1 form a loosely packed aggregate that is similar in size to the results obtained by SANS. At 45 °C, a more collapsed and densely packed ELK1 aggregate is observed. The presence of more collapsed and denser aggregates of ELK1.

Supplementary Section 11. Cytotoxicity evaluation in function of GO concentration

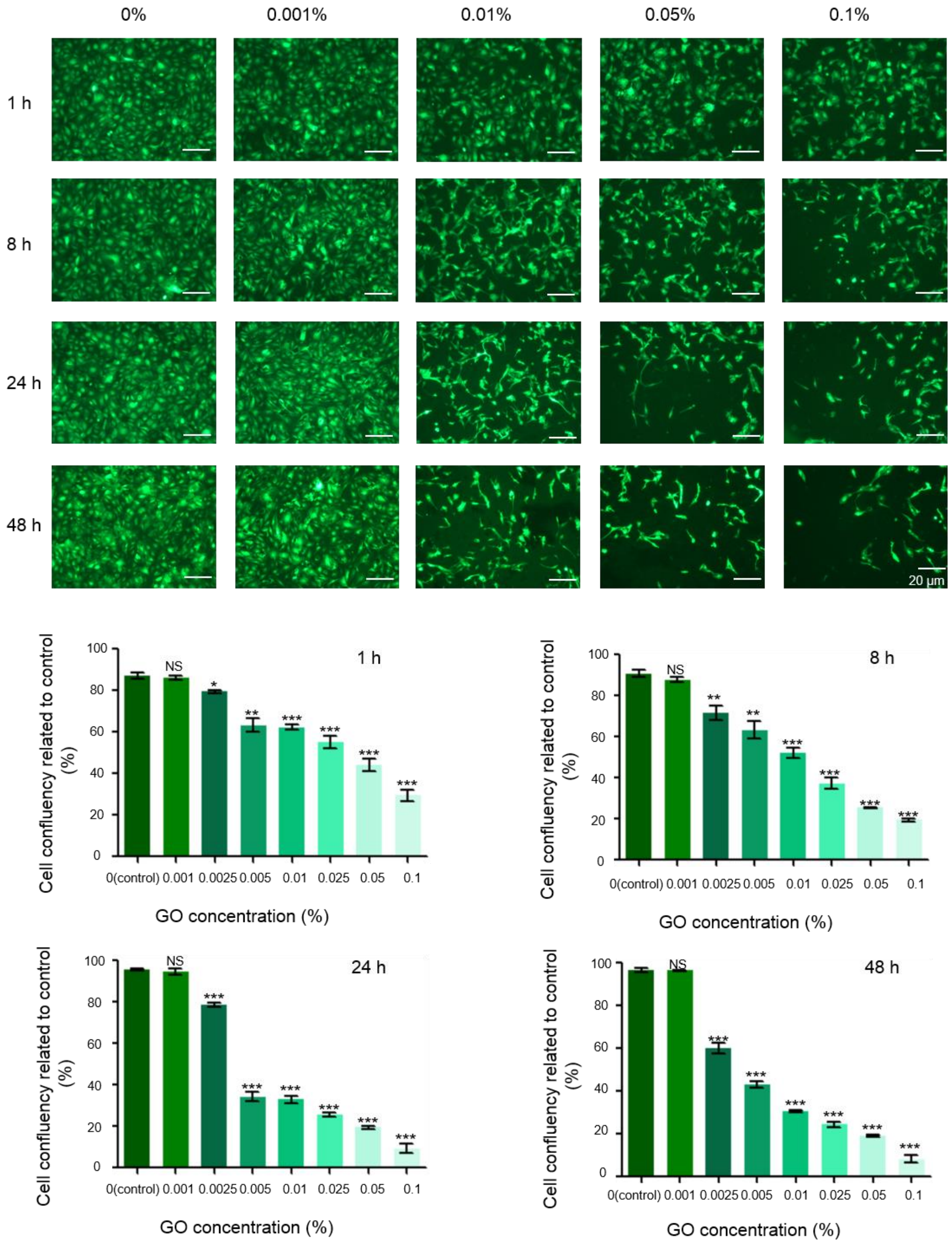

Supplementary Figure 14. Fluorescent images and confluency were achieved at 1, 8, 24 and 48 hr to assess the cytotoxicity of GO by conducting >95% confluent GFP-hUVECs using media containing

varying GO concentrations (0, 0.001, 0.0025, 0.005, 0.01, 0.25, 0.05 and 0.1%). ±*s.d. for n=3. *p< 0.05. NS=no significance.*

Supplementary Section 12. Cytotoxicity evaluation of ELK1-GO degradation products

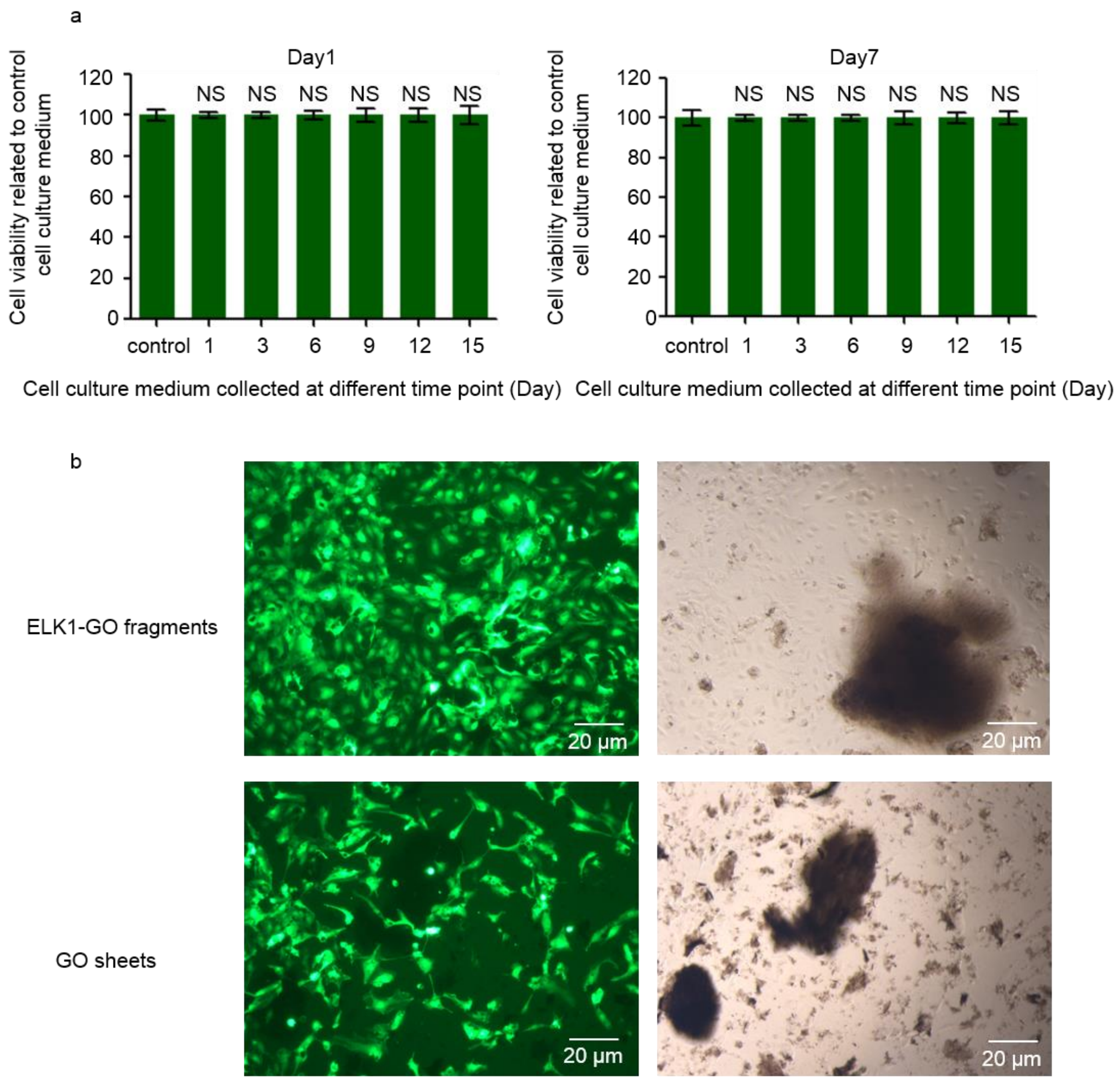

Supplementary Figure 15 a. Confluency achieved at Day 1 and Day 7 by conducting >95% confluent GFP-hUVECs using extracted media co-cultured with ELK1-GO for different durations. **12b.** Fluorescent (left) and bright field (right) images of the same view taken from conducting >95% confluent GFP-hUVECs with ELK1-GO fragments and GO sheets only at similar concentration. ±*s.d. for n=3. *p< 0.05. NS=no significance.*

Supplementary Section 13. Chalkley Score

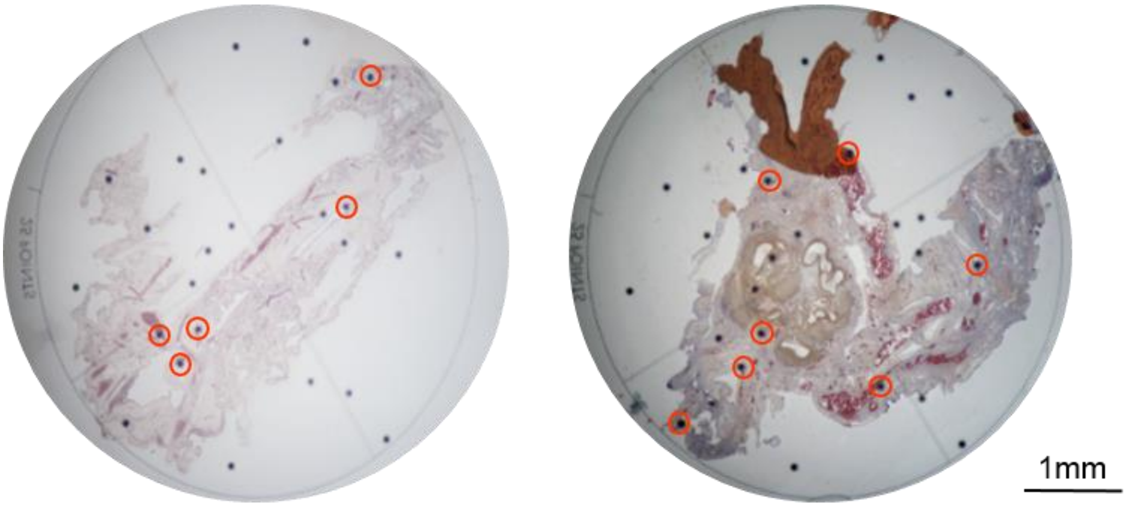

Blank ELK1-GO

Supplementary Figure 16. Quantification of blood vessel formation after implantation of the ELK1-GO tubes in an *ex vivo* preclinical chick chorioallantoic membrane (CAM) model by Chalkley count using a digital overlay. A digital grid that contained 25 random dots was aligned in 3-5 “hotspot” ROIs (areas covering the most blood vessels in the CAM sample tissue). The digital grid was rotated until the most dots landed on the positively stained vessels. The blank sample exhibited a Chalkley count of 5 while the ELK1-GO sample displayed a Chalkley count of 7 (shown by the red circles).

Supplementary Section 14. Statistical analysis of nanotensile mechanical tests

Tensile tests were performed on three kinds of different tube samples: 2% ELK1-0.05% GO, 2% ELK1-0.10% GO, and 2% ELK1-0.15% GO samples to test if the mechanical properties changed with increasing concentration of GO. 40 μL ELK1 solution was added into 200 μL GO solution in a well of 48-well Petri dish. Rectangular membranes were cut about 10 min after their preparation (Supplementary Figure 17). Ten samples for each composition were tested. Since the core of these structures is made of GO, density was assumed to be equal to 1.8 g/cm^3 based on previous studies[3]. Samples were immediately tested in order to avoid their drying. The main purpose of this test was to characterize the mechanical properties of the bulk material through tensile test. Thus, every type of membrane was tested with a nanotensile machine Agilent Technologies T150 UTM (https://www.agilent.com/home): it is provided with high load resolution (about 50 nN), relatively high maximum load (500 mN) and high displacement resolution (about 0.1 nm).

Membranes were mounted one by one on rectangular paper holders. Both ends of the samples were attached using small amounts of super glue, then each sample holder was mounted in the machine and carefully blocked using two grips. The lateral parts of the paper holder were cut so the machine could perform a traction test and calculate samples mechanical characteristics. Samples had a gauge length of 5 mm and were tested with a strain speed of 0.05%/s. The output of the nanotensile test is a load-displacement curve, from which global properties can be derived. Experimental Young's modulus, strength, strain at break, and toughness modulus were analysed and consequently described by Weibull statistical distribution[4,5].

In the following, the Weibull distribution parameters are obtained by assuming the probability of failure F for a sample of volume V under uniaxial stress σ, and related Young's modulus E, strain at break ε, and toughness modulus T. The probability function is expressed as follows (1) (for x equal to σ, E, ε and T):

$$F(x) = 1 - e^{\left[-\frac{V}{V_0}\left(\frac{x}{x_0}\right)^{\alpha x}\right]} \quad (1)$$

Where x_0 and α_x represent the Weibull's scale and shape parameters, respectively, and V_0 is a unit volume. For a generic quantity *x, x0* is obtained from the y-intercept *βx* of the best fit equation, being *βx* equal to *αx*ln(x0)* (from eq. 1), and has the same unit as *x*. The cumulative probability estimators for experimental tests can be obtained as (2)[4]:

$$F(x_i) = \frac{i-0.5}{N} \qquad (2)$$

Where N is the number of tests. All the experimental quantities are ranked in ascending order.

For each quantity, σ_0 (or E_0, ε_0, T_0) and α_σ (or α_E, α_ε, α_T) are the Weibull's scale and shape parameters respectively. σ_0, E_0, ε_0 and T_0 could be considered also as an index of the mean value of the distribution, while α_σ, α_E, α_ε and α_T are the Weibull modulus.

From the statistical analysis, it is possible to state that the Weibull distribution well describes the mechanical properties of samples, as it is possible to deduce from the high values of R^2. Samples with a concentration of 0.10% of GO show a Young's modulus probability distribution that is smoother than the other types of samples. This means that in this case there is a major probability to have high values of *E*. Thus, it is possible to assert that 0.10% GO samples are stiffer than the others. Apart from the elastic modulus, the other mechanical properties increase when increasing the GO concentration. However, these preliminary results suggest that samples with 0.10% GO could exhibit higher stiffness without compromising its strain at break and dissipated energy.

Supplementary Table 1 | Sample dimensions, referring to Fig. 3c.

	**0.05%**		**0.10%**		**0.15%**	
h[mm]	0.98 ± 0.15		1.42 ± 0.37		1.67 ± 0.48	
l [mm]	5.00		5.00		5.00	
t [mm]	0.03	0.05	0.03	0.05	0.03	0.05
ρ [g/cm^3]	1.8		1.8		1.8	

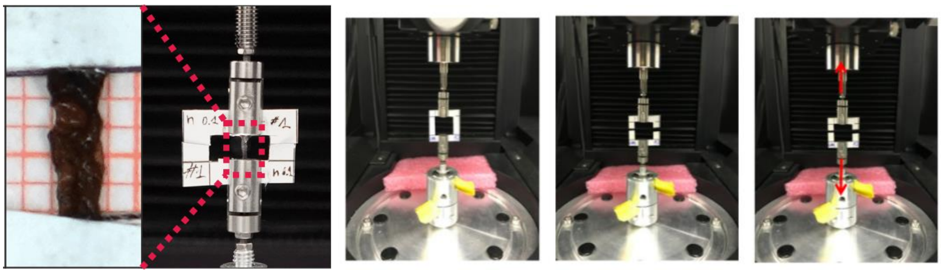

Supplementary Figure 17. Nanotensile test, details of the setup.

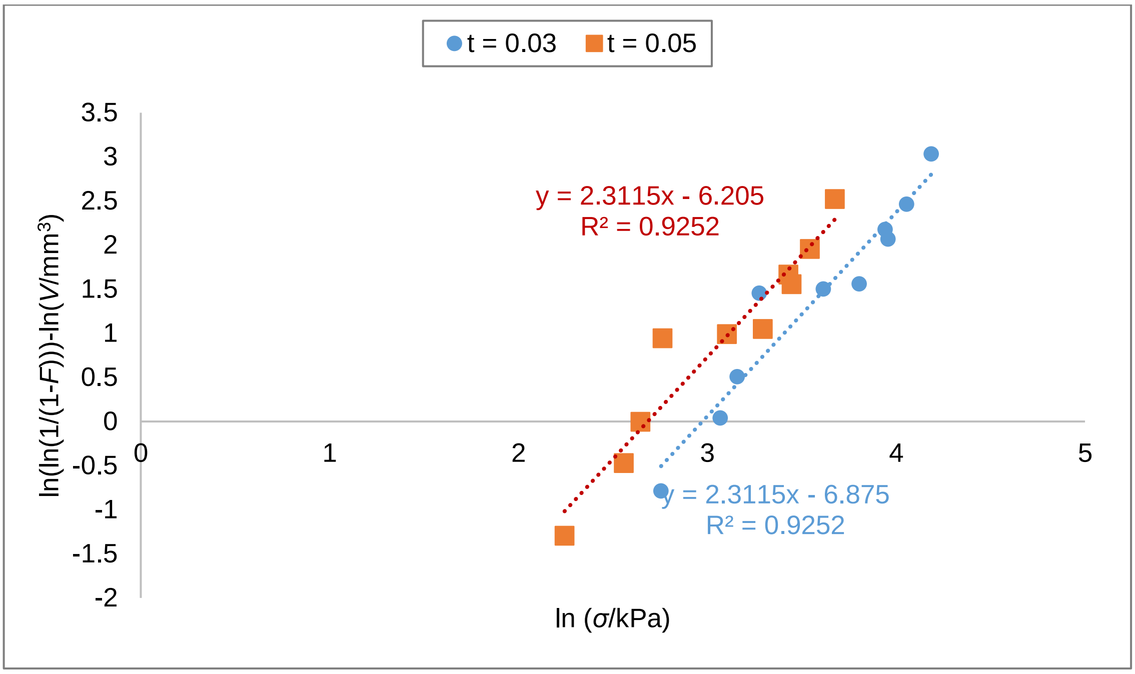

Supplementary Figure 18. Weibull statistics for strength of 0.05% GO samples, by adopting the minimum and maximum thickness (t) values.

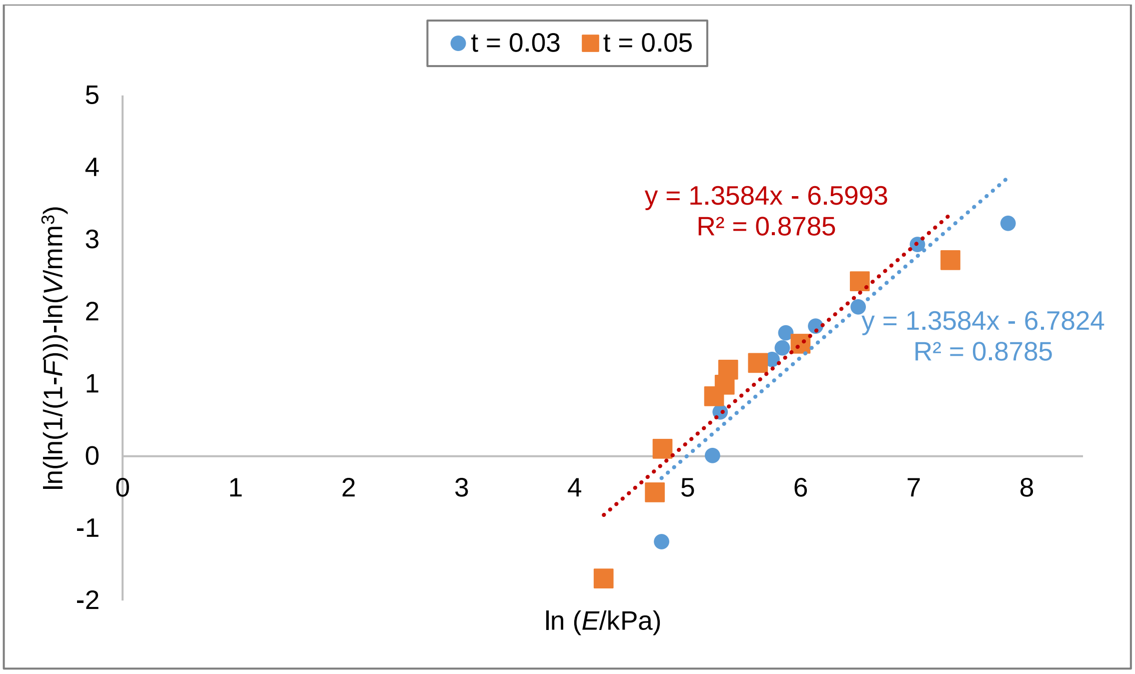

Supplementary Figure 19. Weibull statistics for Young's modulus of 0.05% GO samples, by adopting the minimum and maximum thickness (*t*) values.

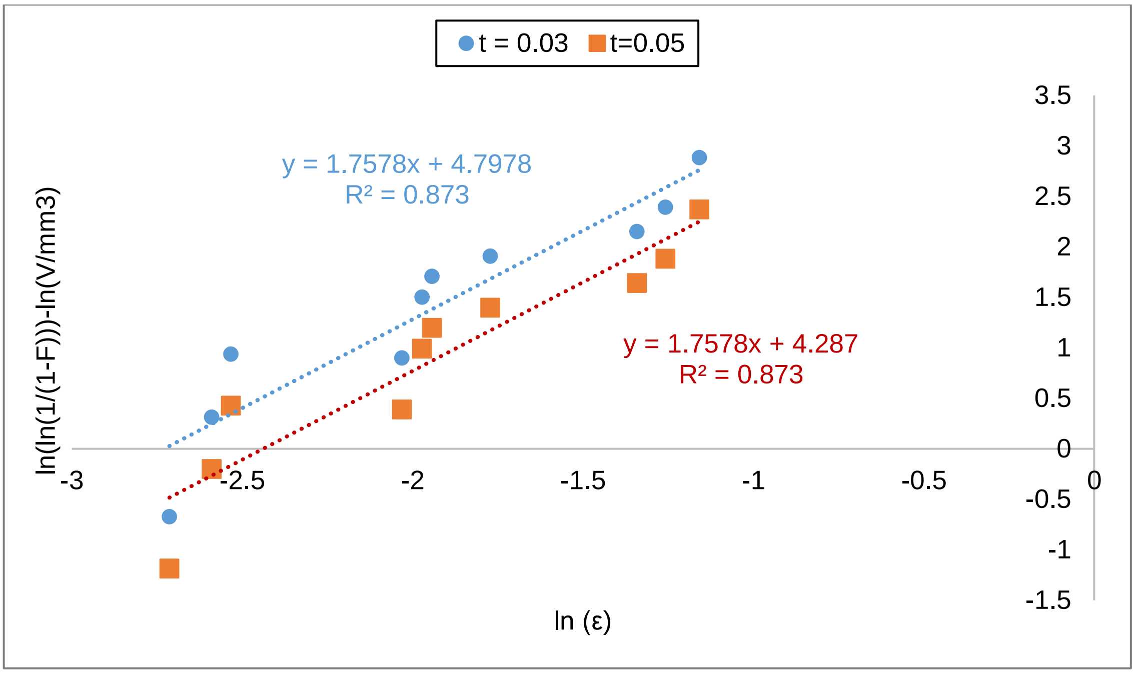

Supplementary Figure 20. Weibull statistics for strain at break of 0.05% GO samples, by adopting the minimum and maximum thickness (*t*) values.

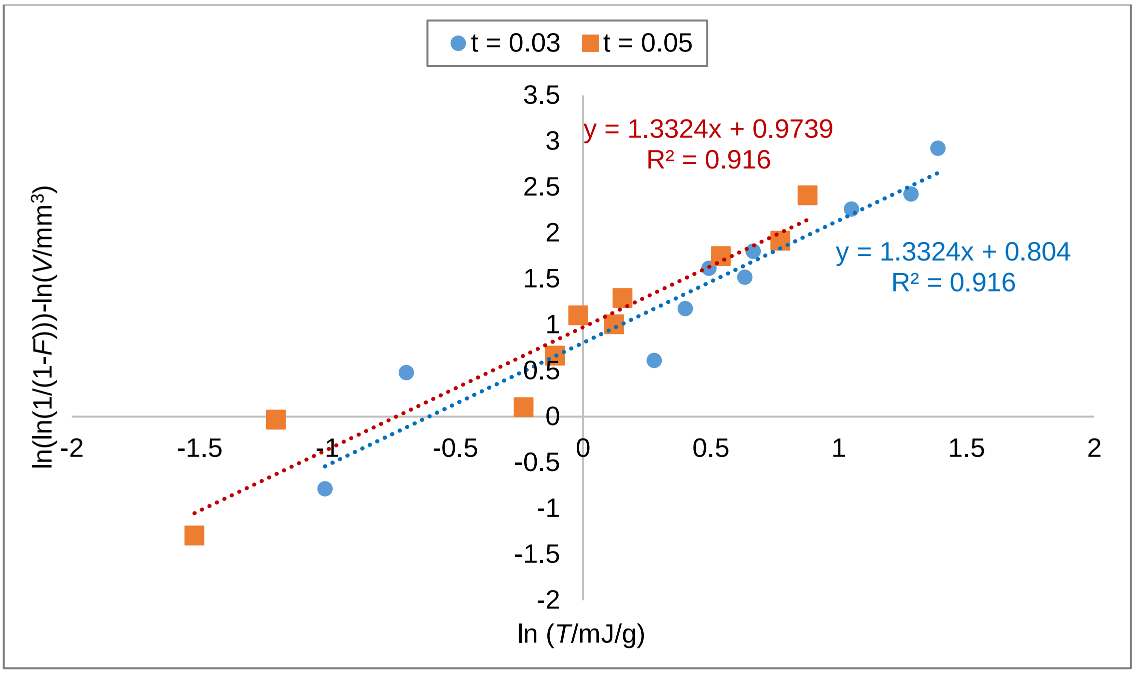

Supplementary Figure 21. Weibull statistics for toughness modulus of 0.05% GO samples, by adopting the minimum and maximum thickness (*t*) values.

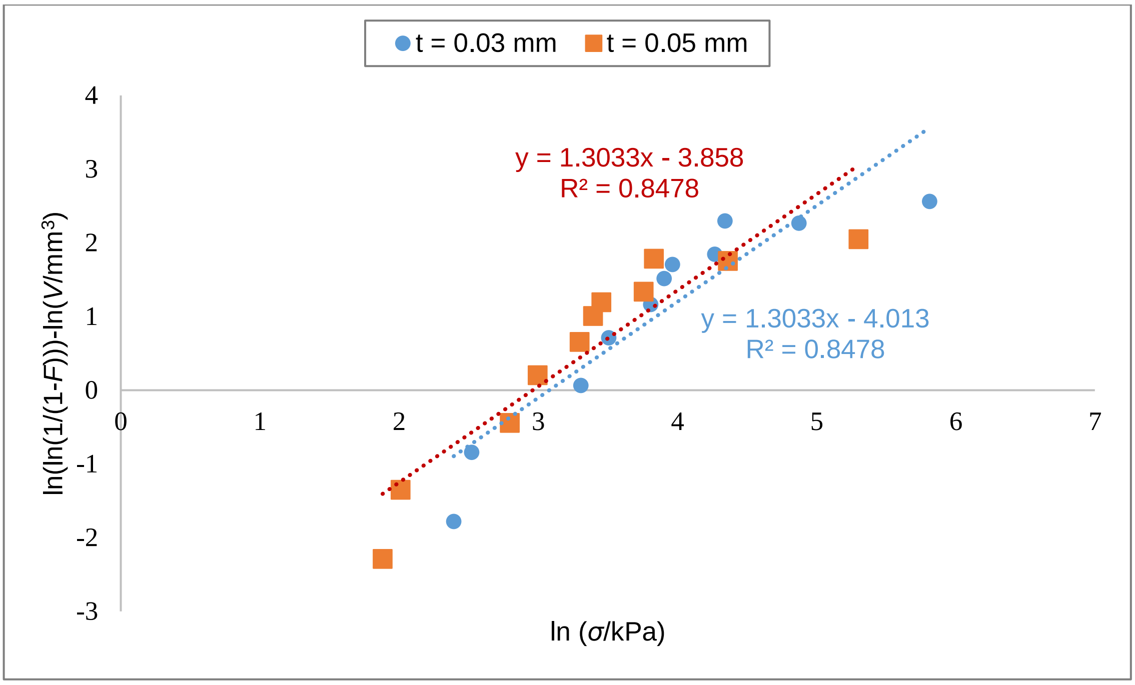

Supplementary Figure 22. Weibull statistics for strength of 0.10% GO samples, by adopting the minimum and maximum thickness (*t*) values.

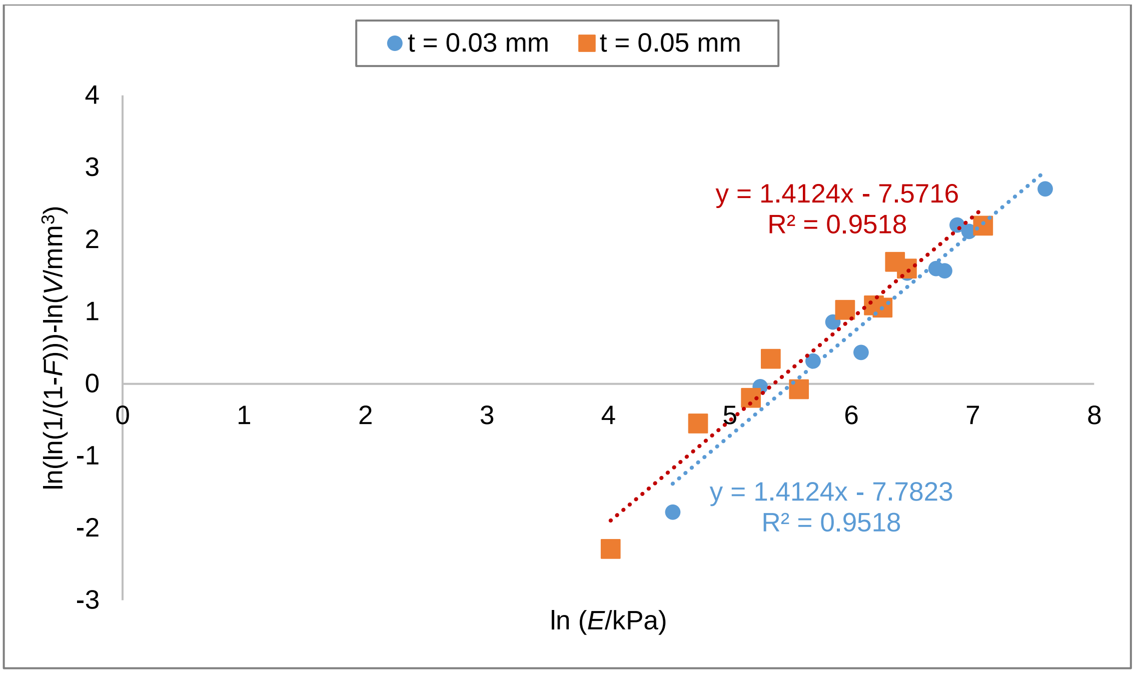

Supplementary Figure 23. Weibull statistics for Young's modulus of 0.10% GO samples, by adopting the minimum and maximum thickness (*t*) values.

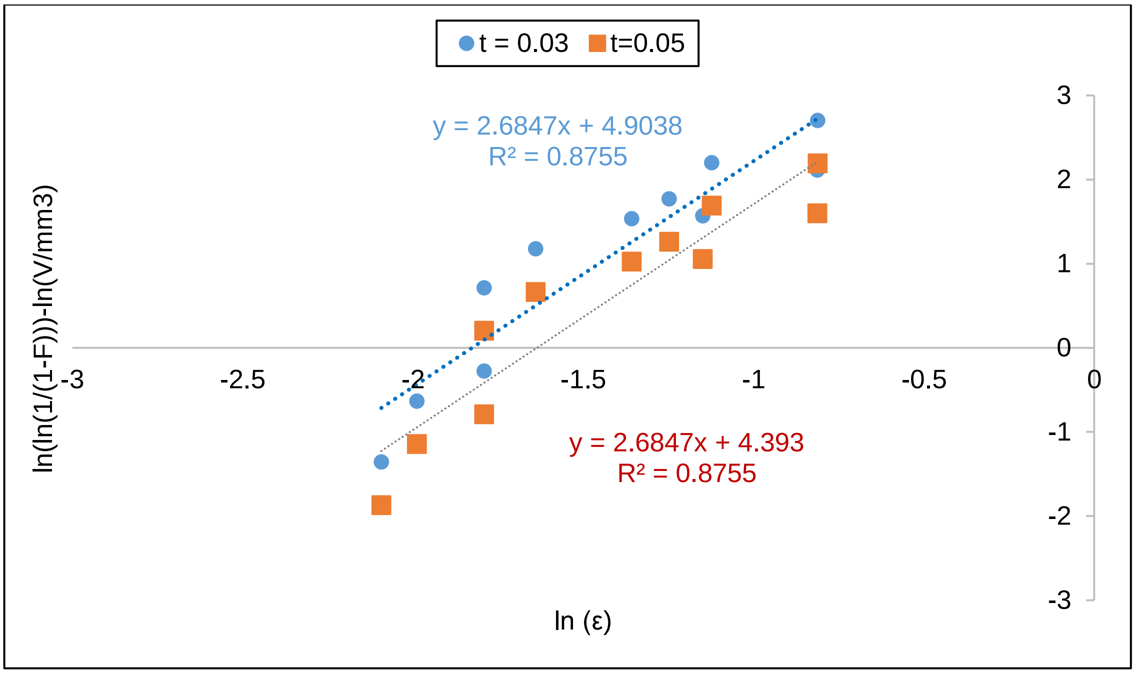

Supplementary Figure 24. Weibull statistics for strain at break of 0.10% GO samples, by adopting the minimum and maximum thickness (*t*) values.

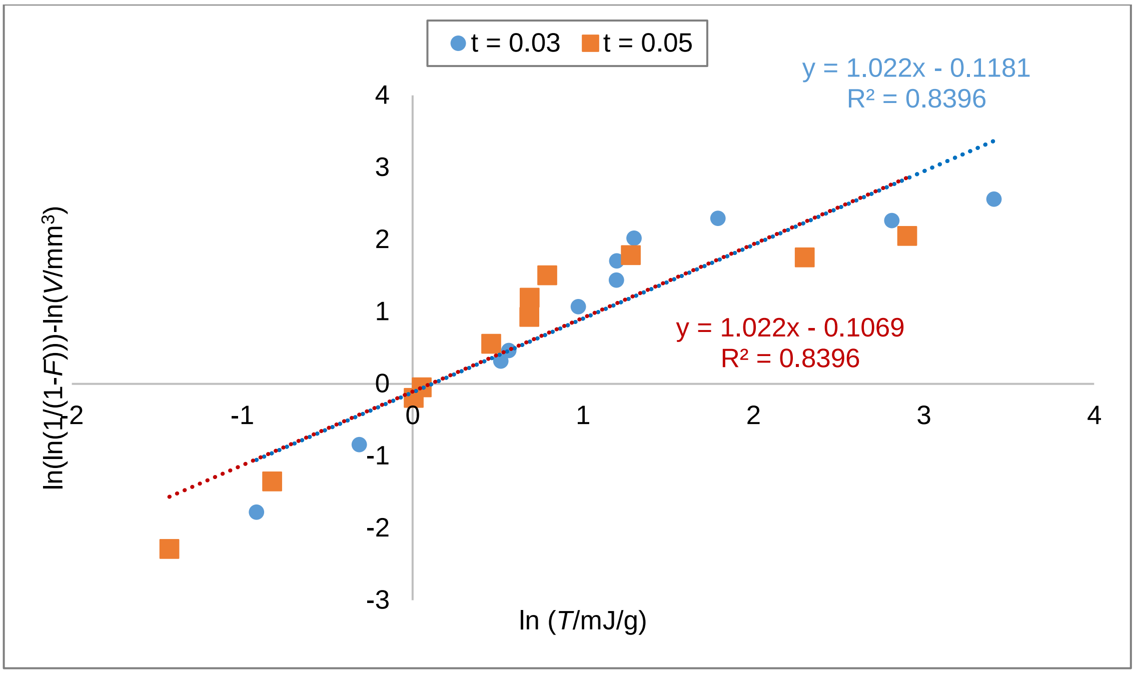

Supplementary Figure 25. Weibull statistics for toughness modulus of 0.10% GO samples, by adopting the minimum and maximum thickness (*t*) values.

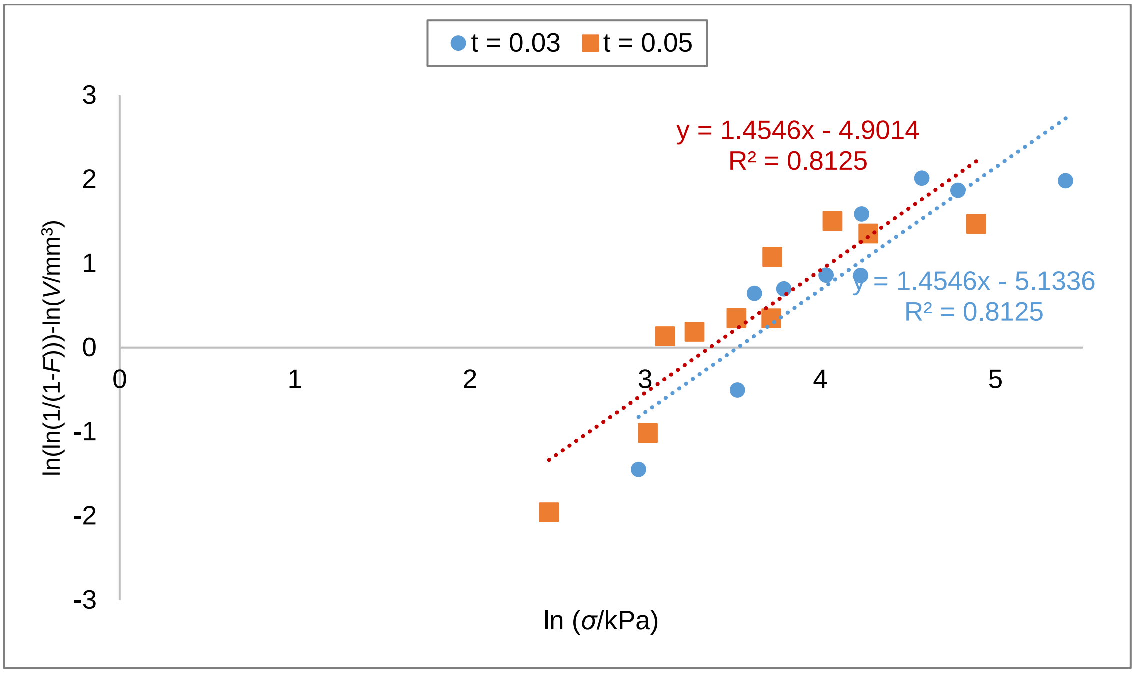

Supplementary Figure 26. Weibull statistics for strength of 0.15% GO samples, by adopting the minimum and maximum thickness (*t*) values.

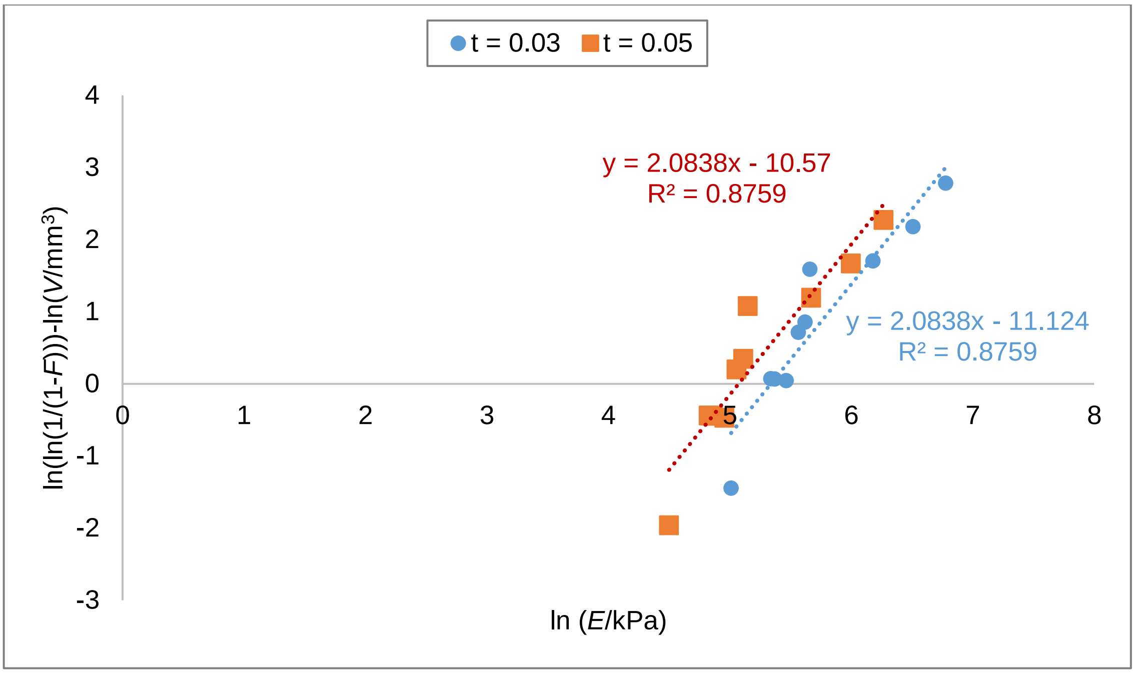

Supplementary Figure 27. Weibull statistics for Young's modulus of 0.15% GO samples, by adopting the minimum and maximum thickness (t) values.

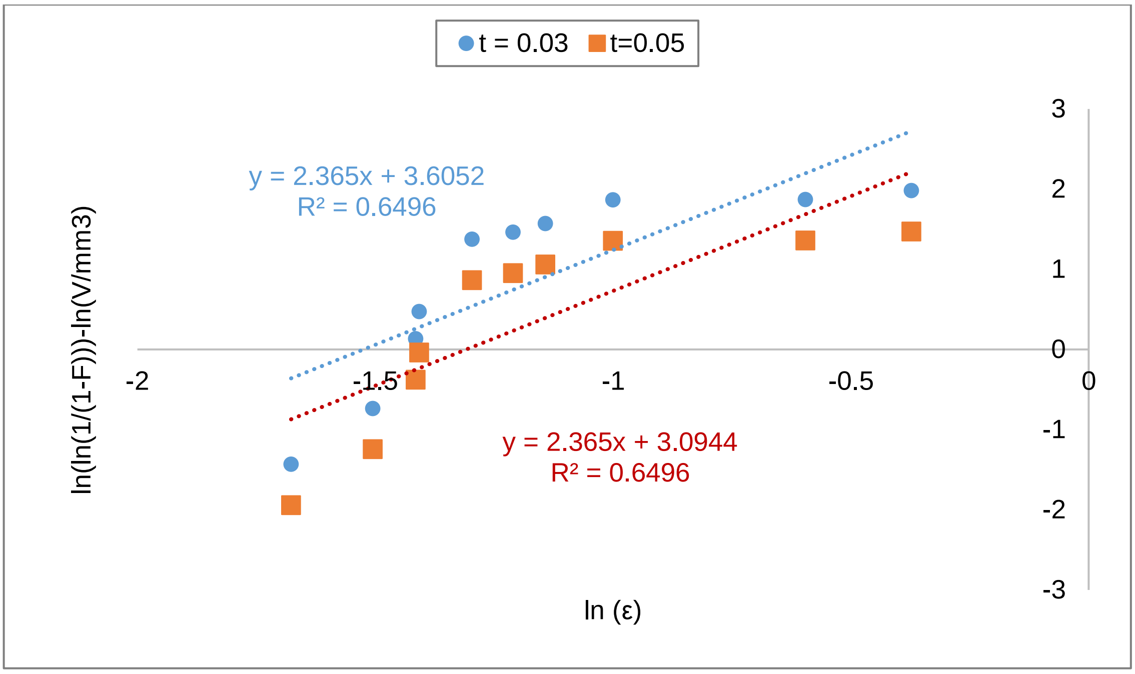

Supplementary Figure 28. Weibull statistics for strain at break of 0.15% GO samples, by adopting the minimum and maximum thickness (t) values.

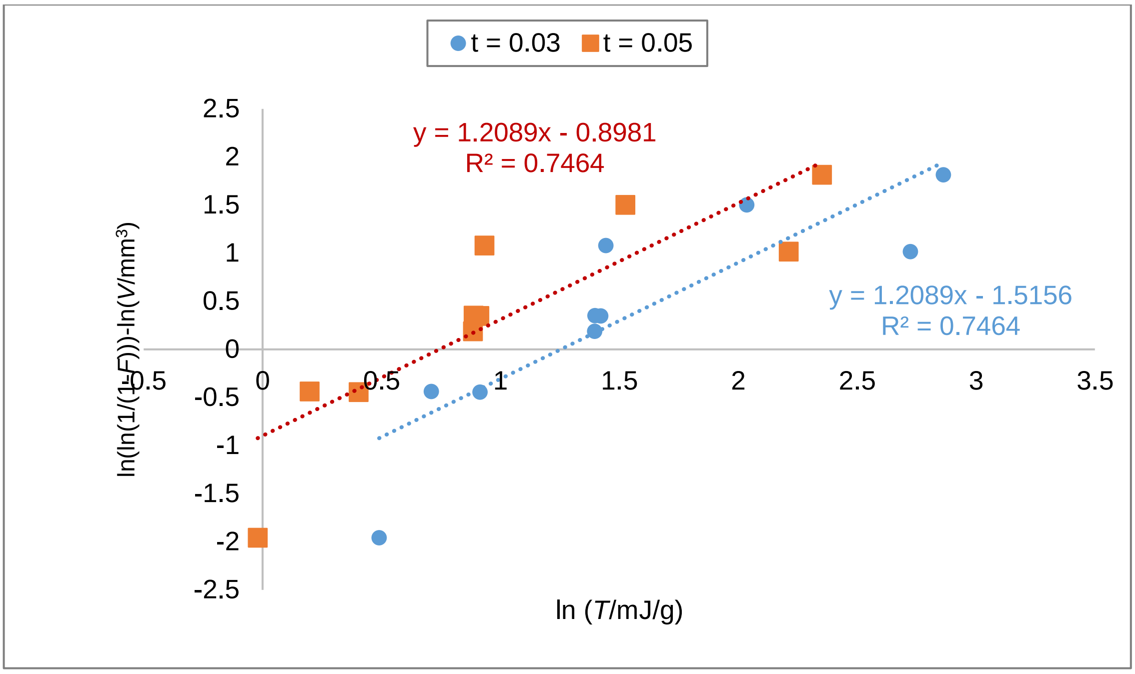

Supplementary Figure 29. Weibull statistics for toughness modulus of 0.15% GO samples, by adopting the minimum and maximum thickness (*t*) values.

Supplementary Table 2 | Weibull statistics parameters for the strength of samples, obtained from the best fit curves. $\sigma_{0\,\mathrm{max}}$ and $\sigma_{0\,\mathrm{min}}$ refer to the minimum and maximum thickness *t* respectively. α and β are respectively the gradient and the intercept of the interpolant straight lines.

	$\sigma_{0\,max}$ [kPa]	$\sigma_{0\,min}$ [kPa]	α_σ	β_σ t = 0.03	β_σ t = 0.05	R^2
**0.05%**	19.58	14.65	2.31	6.88	6.21	0.93
**0.10%**	21.74	19.30	1.30	4.01	3.86	0.85
**0.15%**	34.10	29.07	1.45	5.13	4.90	0.81

Supplementary Table 3 | Weibull statistics parameters for the Young's modulus of samples, obtained from the best fit curves. $E_{0\,\mathrm{max}}$ and $E_{0\,\mathrm{min}}$ refer to the minimum and maximum thickness *t* respectively. α and β are respectively the gradient and the intercept of the interpolant straight lines.

	$E_{0\,max}$ [kPa]	$E_{0\,min}$ [kPa]	α_E	β_E t = 0.03	β_E t = 0.05	R^2
**0.05%**	147.37	128.78	1.36	6.78	6.60	0.88
**0.10%**	247.15	212.90	1.41	7.78	7.57	0.95
**0.15%**	208.16	159.57	2.08	11.12	10.57	0.88

Supplementary Table 4 | Weibull statistics parameters for the strain at break of samples, obtained from the best fit curves. $\varepsilon_{0\ max}$ and $\varepsilon_{0\ min}$ refer to the minimum and maximum thickness t respectively. α and β are respectively the gradient and the intercept of the interpolant straight lines.

	$\varepsilon_{0\ max}$ [-]	$\varepsilon_{0\ min}$ [-]	α_ε	β_ε t = 0.03	β_ε t = 0.05	R^2
**0.05%**	0.07	0.09	1.76	4.80	4.29	0.87
**0.10%**	0.16	0.19	2.68	4.90	4.39	0.88
**0.15%**	0.22	0.27	2.37	3.61	3.09	0.65

Supplementary Table 5 | Weibull statistics parameters for the toughness modulus of samples, obtained from the best fit curves. $T_{0\ max}$ and $T_{0\ min}$ refer to the minimum and maximum thickness t respectively. α and β are respectively the gradient and the intercept of the interpolant straight lines.

	$T_{0\ max}$ [mJ/g]	$T_{0\ min}$ [mJ/g]	α_T	β_T t = 0.03	β_T t = 0.05	R^2
**0.05%**	0.55	0.48	1.33	-0.80	-0.97	0.92
**0.10%**	1.12	1.11	1.02	0.12	0.11	0.84
**0.15%**	3.50	2.10	1.21	1.12	0.90	0.75

Experimental results obtained from tensile tests

Supplementary Table 6 | Mechanical tests results for samples with 0.05% of GO.

	E_{max} [kPa]	E_{min} [kPa]	σ_{max} [kPa]	σ_{min} [kPa]	ε_m [-]	ε_u [-]	T_{max} [mJ/g]	T_{min} [mJ/g]
**Test 1**	461.63	276.98	23.51	14.11	0.21	0.28	1.95	1.17
**Test 2**	117.89	70.74	44.87	26.92	0.28	0.31	3.61	2.17
**Test 4**	198.36	119.02	21.49	12.90	0.13	0.17	1.32	0.79
**Test 5**	185.30	111.18	57.66	34.60	0.23	0.26	4.01	2.41
**Test 6**	343.85	206.31	37.11	22.27	0.11	0.14	1.49	0.90
**Test 7**	673.10	403.86	52.33	31.40	0.07	0.13	1.88	1.13
**Test 9**	1137.03	682.22	26.43	15.86	0.05	0.07	0.50	0.30
**Test 10**	354.08	212.45	65.76	39.45	0.13	0.14	2.86	1.72
**Test 11**	313.29	187.98	15.72	9.43	0.07	0.08	0.36	0.22
**Test 12**	2533.44	1520.06	51.47	30.88	0.06	0.08	1.64	0.98

Supplementary Table 7 | Mechanical tests results for samples with 0.10% of GO.

	E_{max} [kPa]	E_{min} [kPa]	σ_{max} [kPa]	σ_{min} [kPa]	ε_m [-]	ε_u [-]	T_{max} [mJ/g]	T_{min} [mJ/g]
**Test 1**	190.71	114.42	49.59	29.75	0.24	0.29	3.67	2.20
**Test 2**	1063.90	638.34	27.31	16.39	0.15	0.44	1.76	1.06
**Test 3**	964.28	578.57	45.10	27.06	0.03	0.33	3.31	1.98
**Test 4**	870.28	522.17	334.08	200.45	0.22	0.32	30.36	18.21
**Test 6**	437.63	262.58	12.46	7.48	0.13	0.17	0.73	0.44
**Test 7**	810.44	486.26	71.44	42.87	0.11	0.12	2.64	1.59
**Test 8**	347.09	208.25	52.71	31.63	0.15	0.19	3.31	1.99
**Test 9**	1991.04	1194.62	130.71	78.43	0.08	0.44	16.66	10.00
**Test 10**	639.44	383.67	76.94	46.16	0.18	0.26	6.00	3.60
**Test 11**	294.72	176.83	33.34	20.01	0.12	0.17	1.68	1.01
**Test 12**	92.73	55.64	10.94	6.56	0.12	0.14	0.40	0.24

Supplementary Table 8 | Mechanical tests results for samples with 0.15% of GO.

	E_{max} [kPa]	E_{min} [kPa]	σ_{max} [kPa]	σ_{min} [kPa]	ε_m [-]	ε_u [-]	T_{max} [mJ/g]	T_{min} [mJ/g]
**Test 1**	207.86	124.72	37.52	22.51	0.26	0.27	2.03	1.22
**Test 2**	286.85	172.11	44.40	26.64	0.29	0.37	4.04	2.42
**Test 3**	261.16	156.70	119.87	71.92	0.39	0.55	17.52	10.51
**Test 4**	236.31	141.79	221.89	133.13	0.68	0.69	15.23	9.14
**Test 5**	670.22	402.13	69.22	41.53	0.12	0.19	4.24	2.54
**Test 6**	877.23	526.34	97.59	58.55	0.20	0.30	7.66	4.60
**Test 7**	276.14	165.68	68.86	41.32	0.17	0.22	4.15	2.49
**Test 8**	482.86	289.72	56.48	33.89	0.18	0.24	4.05	2.43
**Test 9**	150.05	90.03	19.35	11.61	0.29	0.32	1.63	0.98
**Test 11**	214.86	128.91	34.04	20.42	0.19	0.24	2.50	1.50

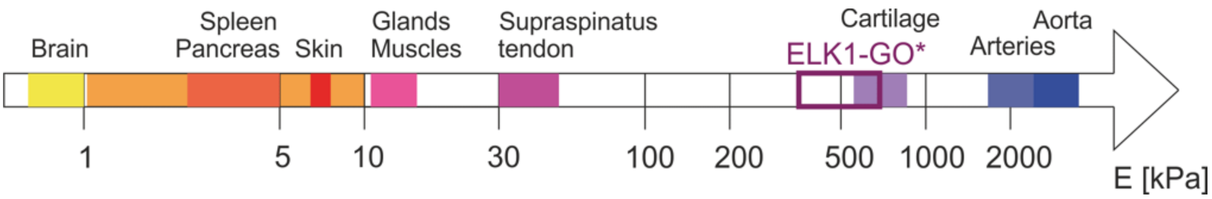

Supplementary Figure 30. (*) Mean values of the Young's modulus for ELK1-GO samples ($E_{0.05\%}$ = 561 kPa, $E_{0.10\%}$ = 683 kPa, $E_{0.15\%}$ = 356 kPa), described by a Weibull distribution with scale parameters E_0 reported in Fig.3c compared with human native tissues[6–11].

Supplementary Section 15. Zeta potential (ζ) of ELK1 and GO solutions at different pHs

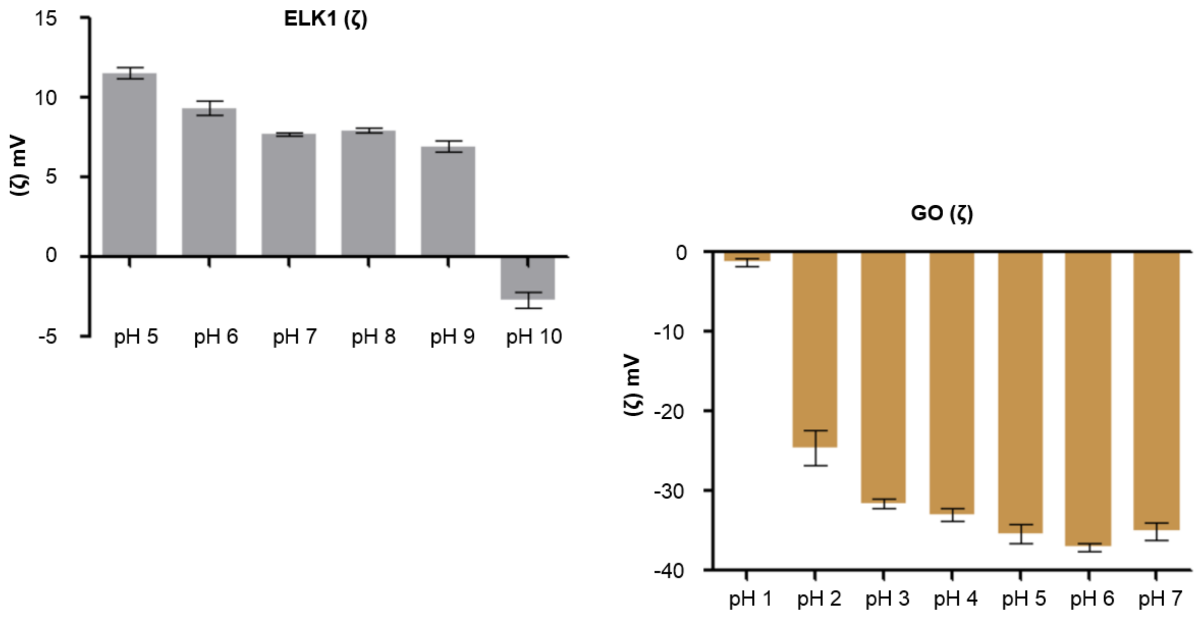

Supplementary Figure 31. Zeta potential of ELK1 and GO**.** Measurements were performed at 30 °C in MilliQ water. ELK1 molecules were found to be positively charged at pH values from 5 to 9, but negatively charged at pH 10. GO was found to be negatively charged at all measured pHs.

Supplementary Section 16. Effect of ionic strength on the formation of ELK1-GO system

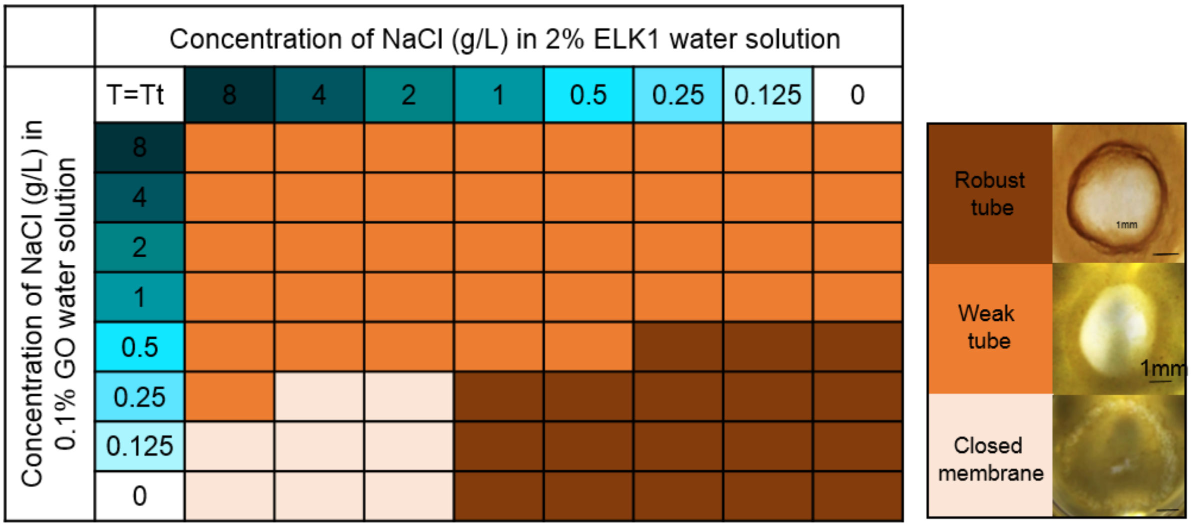

Supplementary Figure 32. Well-defined ELK1-GO tubes can be formed with concentrations of NaCl no more than 1g/L.

Supplementary Section 17. Polydispersity index (PDI) of dynamic light scattering (DLS)

Supplementary Table 9 | PDI of ELK1-GO's DLS

Temperature (°C)	PDI
4	0.39±0.14
30	0.52±0.26
45	0.55±0.09

Supplementary Section 18. Computer modelling.

Molecular models of simulated systems

Our modelling was focused on the interactions between Graphene Oxide (GO) and protein molecules (ELK1) in water. The instrument of choice was classical all-atom Molecular Dynamics (MD) because it allows for precise definition of the system composition and very detailed examination of its behavior at considerable (hundreds of nanoseconds) time.

Therefore, we assume the ELK_{1-1} (VPGIG VPGIG VPGKG VPGIG VPGIG) are able to reveal the functional groups contributing to the GO–ELK1 interactions, it is enough to simulate a single repeat block. This makes the system of interest readily feasible for meaningful MD. The GO sheets are of µm-s size, which makes their edge length negligibly small compared to their area. We, therefore, used periodic boundary conditions (PBC) to model an "infinitely large" sheets. The sheets homogenously oxidized across the plane. According to the known C: O: H ratio 2:1:1, each second C atom holds a hydroxyl group. Thus, in simulation we can use sheets of the same chemical composition but reduced in size without loss of accuracy, with the help of PBC.

Examined molecular systems and simulation details

MD simulations were performed using free open source software GROMACS 5.1 [15] on the HPC Midlands supercomputer cluster. The initial configuration of ELK_{1-1} was generated from the sequence using an on-line platform implementing the I-TASSER based algorithms for protein structure predictions[16]. The termini were charged and the lysine residues were protonated; Cl^- was chosen as the counter-ion for overall system electroneutrality. The initial GO structure was reconstructed in two stages: first, a graphene sheet was generated using the VMD Nanotube builder plugin[17], then OH groups were attached to randomly chosen atoms using own scripts. Finally, three sheets were placed next to each other to form a stack mimicking the GO lamellas. According to MD simulations of Shih *et*

al.[18], the C – C distance between neighbouring sheets was set to 0.8 nm, so that a layer of water molecules can be fitted between the sheets.

Following previous studies of graphene and graphene oxide[18,19], the OPLS-AA forcefield[20] was employed together with SPC/E water model. In GO, the non-oxidized C atoms were described by the #147 atomtype (naphthalene rings junction) and had no partial charge. For C–O–H groups, the parameters of triatomic alcohols were adopted: atomtypes #175, #171, #172, and partial charges 0.265, -0.73, and 0.465, respectively.

The simulation parameters, common for all the simulated systems, are as follows: 3D periodic boundary conditions; Berendsen thermostat with time constant 2 ps, applied individually to protein and water; particle mesh Ewald method for computing electrostatic interactions; cut-off of van der Waals interactions at 1 nm; constraints on all bonds (LINCS algorithm).

Two kinds of systems were considered:

1) A single ELK_{1-1} peptide in water was simulated with the aim to reach the equilibrium conformation at the given temperature, Supplementary Figure 33a. The parameters were: cell size 8x8x8 nm^3, run time 440 ns, temperatures 4°C, 30 °C, 45 °C, pressure of 1 bar, maintained with Berendsen barostat with time constant 1.5 ps. The initial peptide conformation was generated using I-TASSER.
2) Three ELK_{1-1} peptides in water with an 8x8 nm^2 GO stack were simulated to identify functional groups responsible for the interactions, Supplementary Figure 33b. The parameters were: cell size 8x8x16 nm^3, run time 100 ns, temperatures 30 °C, and constant volume. The GO stack was fixed during the simulations, only H atoms were allowed to move. Two initial configurations were used for simulation: i) the ELK_{1-1} peptides distributed in solution, and ii) the ELK_{1-1} peptides placed near the GO surface, Supplementary Figure 33c.

Simulation results for an ELK1-1 peptide in water

The secondary structure of an ELK1-1 peptide was computed using DSSP 2.0.4 software[21], Supplementary Figure 34. The structure does not change considerably during the last 40 ns of the runs, thus it can be considered as converged. No alpha-helices were identified, which agrees with the experimental results (Supplementary Figure 8). Although at equilibrium, the secondary structure does change slowly, it can be seen that the conformational transitions happen at the time scale of hundreds of nanoseconds – microseconds.

The hydrophilic and hydrophobic solvent-accessible surface areas (as computed by VMD on the last 40 ns of the trajectory) at 4 °C were equal to 7.4 nm^2 and 12.3 nm^2 , respectively, while at 30 °C the values were 6.5 nm^2 and 13.8 nm^2, respectively. The values at 45 °C were similar to those at 30 °C. Therefore, the ratio of hydrophilic/hydrophobic parts of the protein surface was approximately the same for all three temperatures, showing no considerable change of hydrophobicity occurring.

Simulation results for three ELK1-1 peptides *with GO*

First, consider the runs where ELK_{1-1} peptides were initially located near the GO surface. The aggregate of peptides did not detach from GO during the whole simulation period. It stayed connected to GO by several hydrogen bonds (Supplementary Figure 35b). This proves that MD correctly reproduces the presence of strong interactions between the peptides and GO.

Now, turn to the runs, started with peptides in the bulk solution. One peptide became adsorbed on the GO surface during the simulation. During 100 ns, sticking of one peptide molecule to another, previously adsorbed on GO, occurred. Thus, we extended the run for additional 100 ns, but no changes occured. The final result was an aggregate of three peptides attached to GO by several hydrogen bonds.

Visual examination of the trajectories clearly shows the main factor ensuring the adhesion of the ELK_{1-1} peptides to GO. It is the protonated amino group of lysine residues, which forms hydrogen bonds with the OH groups of GO. The rest of the peptide usually almost does not make contacts with GO and is separated from it by water, which is possible because the lysine's amino group is bonded to a long hydrocarbon chain that can stretch out. Occasionally, the –CO–NH– peptide bonds of the protein's backbone also make contacts with GO and form hydrogen bonds, but this is usually not the main factor. The average numbers of hydrogen bonds between the species (in the runs with initially adhered

peptides) are listed in Supplementary Figure 35a and the typical snapshots from the simulations are shown in Supplementary Figure 35b. Further, the charged termini (COO^- and NH_3^+) also actively take part in adsorption.

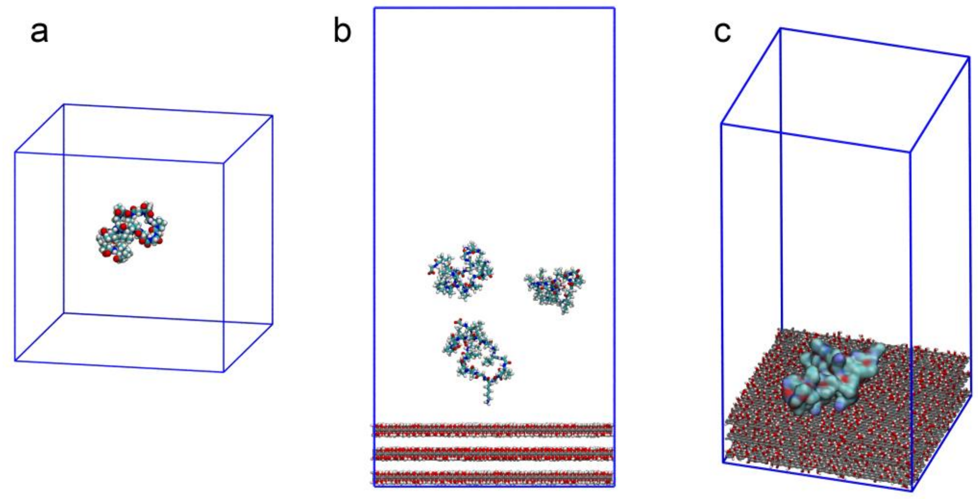

Supplementary Figure 33. Initial configurations for simulated systems. **a.** An ELK1-1 peptide was put in water. This system aimed to simulate the first part including one ELK1-1 peptide in water at 3 different temperatures. **b.** Three ELK1-1 peptides and a stack of GO sheets in water together before running the simulation to identify interactions between them. This system aimed to simulate the second part. **c.** To check the dependence of MD results from initial conditions, three ELK1-1 peptides were initially located near the GO surface to test whether the interaction between ELK1-1 and GO is strong enough to keep this binding status. The system corresponds to the second part, too. The aggregate did not detach from the GO during the whole simulation period being connected with GO by several hydrogen bonds.

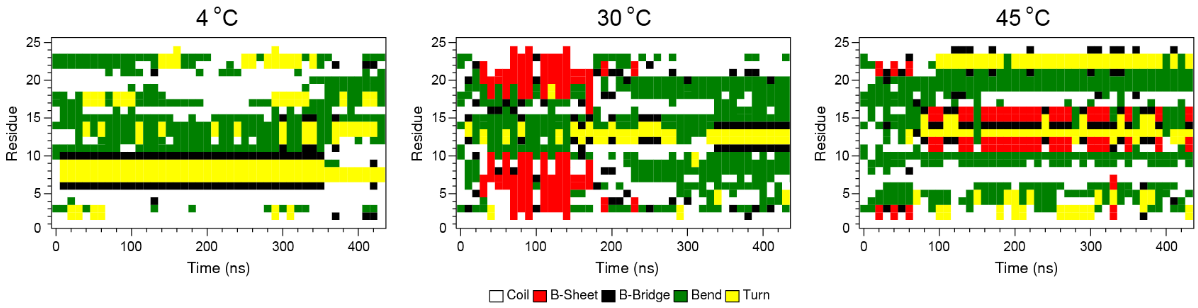

Supplementary Figure 34. Investigation of changes in the secondary structure of an ELK1-1 peptide alone in water at different temperatures. The simulation did not show a similar structure change as that of the full-length ELK1.

a

Types of H-bonds	Number of H-bonds
Lysine–GO	2.68
Peptide bonds–GO	2.47
Termini–GO	0.28

b

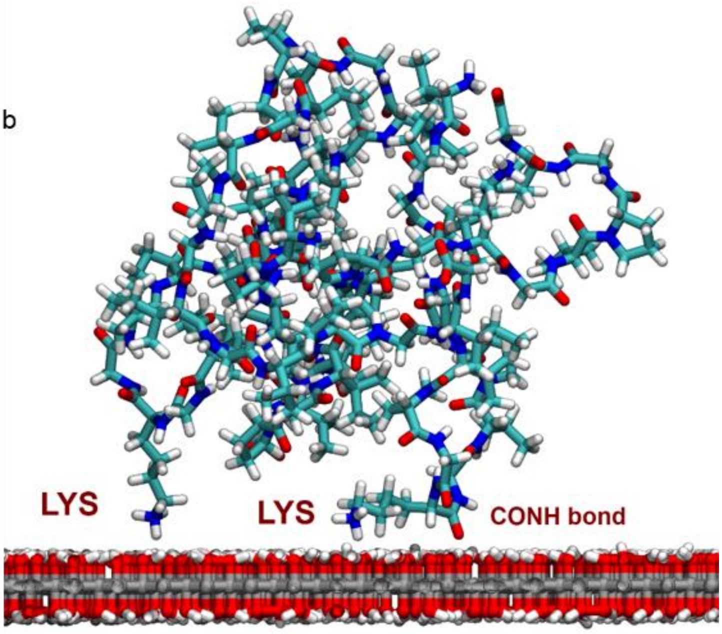

Supplementary Figure 35. Hydrogen bond interactions between ELK1-1 and GO. **a.** The average number of hydrogen bonds between ELK1-1-GO. **b.** Instantaneous snapshot from MD simulations of three ELK1-1 peptides with GO indicating the groups responsible for hydrogen bonding.

Supplementary References

1. SasView. SasView for small angle scattering analysis. Available at: https://www.sasview.org/. (Accessed: 19th August 2018)

2. Berke, B., Czakkel, O., Porcar, L., Geissler, E. & László, K. Static and dynamic behaviour of responsive graphene oxide–poly(N-isopropyl acrylamide) composite gels. *Soft Matter* **12,** 7166–7173 (2016).

3. Dikin, D. A. *et al.* Preparation and characterization of graphene oxide paper. *Nature* **448,** 457–460 (2007).

4. Pugno M., N. & Ruoff S., R. Nanoscale Weibull Statistics for Nanofibers and Nanotubes. *J. Aerosp. Eng.* **20,** 0–4 (2007).

5. W. Weibull. A Statistical Distribution Function of Wide Applicability. *J. Appl. Mech.* **18,** 293–297 (1951).

6. Park, D. *et al.* Author Correction: The use of microfluidic spinning fiber as an ophthalmology suture showing the good anastomotic strength control. *Sci. Rep.* **8,** 13293 (2018).

7. Liu, J., Zheng, H., Poh, P. S. P., Machens, H.-G. & Schilling, A. F. Hydrogels for Engineering of Perfusable Vascular Networks. *Int. J. Mol. Sci.* **16,** 15997–6016 (2015).

8. Ling, Y. *et al.* A cell-laden microfluidic hydrogel. *Lab Chip* **7,** 756 (2007).

9. Hasegawa, H. & Kanai, H. Measurement of Elastic Moduli of the Arterial Wall at Multiple Frequencies by Remote Actuation for Assessment of Viscoelasticity. *Jpn. J. Appl. Phys.* **43,** 3197–3203 (2004).

10. Dobrin, P. B. Mechanical properties of arteries. *Physiol. Rev.* **58,** 397–460 (1978).

11. Arda, K., Ciledag, N., Aktas, E., Arıbas, B. K. & Köse, K. Quantitative Assessment of Normal Soft-Tissue Elasticity Using Shear-Wave Ultrasound Elastography. *Am. J. Roentgenol.* **197,** 532–536 (2011).
