# Supplementary material for: Disordered protein-graphene oxide co-assembly and supramolecular biofabrication of functional fluidic devices
Source: Nat Commun. 2020 Mar 4;11:1182. doi: 10.1038/s41467-020-14716-z (PMC7055247; doi:10.1038/s41467-020-14716-z)
Supplement: Supplementary file 2 — Description of Additional Supplementary Files [file 41467_2020_14716_MOESM2_ESM.docx]

**Description of Additional Supplementary Files**

**Supplementary Movie 1.** ELK1-GO system forming first a closed sac when a drop of ELK1 solution is immersed in a larger GO solution and then another one opening upon touching an interface. The video speed has been adjusted as follows: 1-15s = 30%, 15-23s = 2000%, 24-25s = 100%, 25-43s = 50%, and 43-49s = 2000% of original speed.

**Supplementary Movie 2.** ELK1-GO system grown into vertical tubes. The video speed has been adjusted as follows: 0-2s = 1000%, 2-8s = 500%, and 8-10s = 5000% of original speed.

**Supplementary Movie 3.** Making of ELK1-GO tube bridging two surfaces: horizontal growth. The video speed has been adjusted as follows: 0-11s = 100% and 11-19s = 1500% of original speed.

**Supplementary Movie 4.** 3D printing of the ELK1-GO system. The video speed has been adjusted to 150% of the original speed.

**Supplementary Movie 5.** Close-up of fluidic device made by printing/self-assembling the ELK1-GO system showing the flow of water with green food dye. Video is displayed in real time and is looped 4 times.

**Supplementary Movie 6.** Close-up of a bifurcation of a fluidic device made by printing/self-assembling the ELK1-GO system showing the flow of water with green food dye. Video is displayed in real time and is looped 6 times.

**Supplementary Movie 7.** Close-up of a tubular fluidic device made by self-assembling the ELK1-GO system showing the flow of water with green food dye and manually removing the flow. The video speed has been adjusted to 300% of the original speed.

**Supplementary Movie 8.** Close-up of a tubular fluidic device made by self-assembling the ELK1-GO system showing the flow of water with green food dye and self-circulate for 24h without any damage. Video is displayed in real time.
